# Models for total, elastic and diffractive cross sections

**DOI:** 10.1140/epjc/s10052-018-5940-8

**Published:** 2018-06-07

**Authors:** Christine O. Rasmussen, Torbjörn Sjöstrand

**Affiliations:** Theoretical Particle Physics, Department of Astronomy and Theoretical Physics, Lund University, Sölvegatan 14A, 223 62 Lund, Sweden

## Abstract

The LHC has brought much new information on total, elastic and diffractive cross sections, which is not always in agreement with extrapolations from lower energies. The default framework in the Pythia event generator is one case in point. In this article we study and implement two recent models, as more realistic alternatives. Both describe total and elastic cross sections, whereas one also includes single diffraction. Noting some issues at high energies, a variant of the latter is proposed, and extended also to double and central diffraction. Further, the experimental definition of diffraction is based on the presence of rapidity gaps, which however also could be caused by colour reconnection in nondiffractive events, a phenomenon that is studied in the context of a specific model. Throughout comparisons with LHC and other data are presented.

## Introduction

The LHC has provided new information on any number of topics, including total, (differential) elastic and (differential) diffractive cross sections, or $\sigma _{\mathrm {TED}}$ for short. The $\sigma _{\mathrm {TED}}$ kind of quantities cannot be predicted from the QCD Lagrangian, although this is where they have their origin. Therefore $\sigma _{\mathrm {TED}}$ results are often overshadowed by results from the perturbative domain, where comparisons with the Standard Model, and searches for physics beyond it, are more directly related to the underlying theory. Nevertheless, there are good reasons to study the old and new $\sigma _{\mathrm {TED}}$ data now available. One is to assess how well different effective models can describe the data, and implicitly or explicitly pave the way for better models and better understanding, ultimately to form a stronger connection with the underlying QCD theory. Another is that diffractive events form part of the “underlying event” and pileup backgrounds that have a direct impact e.g. on jet energy scales and jet profiles, and thereby on many experimental studies. In this latter aspect they combine with the inelastic nondiffractive events into the overall inelastic event class, with a separation that is far from unambiguous, as we will see. In this article we consider the three simplest diffractive event classes: single, double and central diffraction, corresponding to the dissociation of one, two or zero of the incoming protons, respectively.

**Fig. 1:**
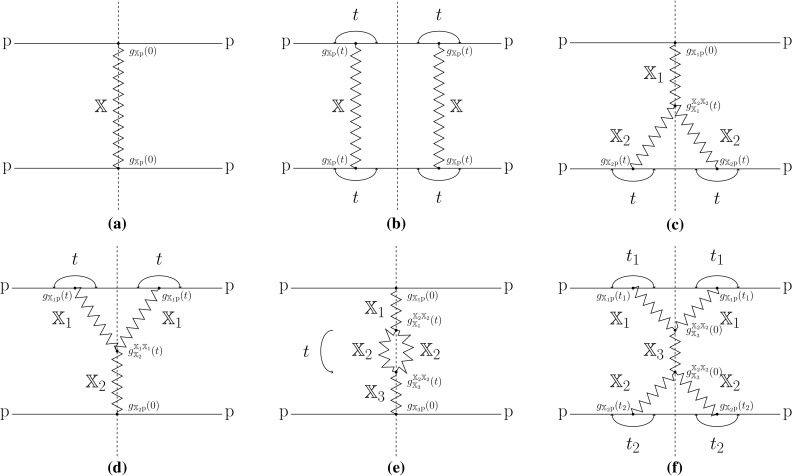
The squared matrix element for the total (**a**), elastic (**b**), single (**c**, **d**), double (**e**) and central (**f**) diffractive cross sections

Historically there are two main approaches to $\sigma _{\mathrm {TED}}$ in hadron–hadron collisions, the diagrammatical and the geometrical, although both aspects may well be represented in a specific model [1–4]. In the diagrammatical approach new effective particles are introduced, specifically the Pomeron(s) $\mathbb {P}$ and Reggeon(s) $\mathbb {R}$, with associated propagators and vertex coupling strengths. A Feynman-diagram-like expansion may be performed into different event classes, with higher-order corrections. A subset of these are shown in Fig. 1, with $\mathbb {X}=\mathbb {P},\mathbb {R}$ and each of the couplings denoted with a *g*. In the diagrammatical approach, the dashed line (the cut) represents the diagram at amplitude level. A cut through a $\mathbb {P}$ or $\mathbb {R}$ thus represent particle formation at amplitude level, while an uncut Pomeron or Reggeon represents an area void of particle production. In a geometrical approach the impact-parameter aspects are emphasized, where diffraction largely is related to peripheral collisions. The analogy with wave scattering theory here is natural, and has given the diffractive event class its name. Diffraction can also be viewed as a consequence of the interaction eigenstates being different from the mass ones [5, 6].

Neither of these approaches address the detailed structure of diffractive events. In olden days, at low energies, a diffractive system was simply viewed as an excited proton state that could decay more-or-less isotropically, a “fireball” [1, 7]. This is clearly not a valid picture for higher-mass diffractive states, where the same kind of longitudinal structure is observed as for nondiffractive ones. The simplest partonic approach would then be for a $\mathbb {P}/\mathbb {R}$ to kick out a single quark or gluon from a proton, giving rise to one or two fragmenting colour strings. The Ingelman–Schlein picture [8] takes it one step further and introduces an internal structure for the $\mathbb {P}$, such that a $\mathbb {P}{\mathrm p}$ collision may be viewed as an inelastic nondiffractive ${\mathrm p}{\mathrm p}$ (or better $\pi ^0{\mathrm p}$) one in miniature. Thereby also hard jet activity and multiparton interactions (MPIs) become possible within a diffractive system, as supported by data.

A key aspect of MPI modelling is the relation to colour reconnection (CR), whereby partons in the final state may be related in colour so as to reduce the total string length relative to naive expectations. This opens for another view on diffraction, where CR can generate rapidity gaps dynamically [9, 10]. Then the diffractive and inelastic nondiffractive event classes have a common partonic origin, and only differ by the event-by-event fluctuations in colour topologies. Even in models that do not go quite as far, the dividing line between the two kinds of events may be fuzzy. This is even more so since the experimental classification in terms of a rapidity gap allows for misidentification in both directions, relative to the classification in a specific model. High-mass diffraction need not give a gap in the central detector, while nondiffractive events by chance (CR or not) can have a large rapidity gap. The classification of each event type in Pythia 8, however, is independent of CR model, such that no double counting occurs on a theoretical level. Each event type has its own specific cross section and, in a combined sample, the mix of event types is based on that. The experimental signature of the events, however, does differ depending on CR model. Thus it is likely that CR models that can give large gaps in nondiffractive events will need a suppression of the diffractive cross sections in order to describe data.

What should now be clear is that description of the $\sigma _{\mathrm {TED}}$ physics, and especially the diffractive part, is too multifaceted to be based purely on analytical calculations. The implementation into Monte Carlo Event Generators is crucial to test different approaches. One of the most commonly used generators is Pythia [11, 12], which by default is based on a rather old diagrammatical “tune” for the $\sigma _{\mathrm {TED}}$ issues [13], combined with an Ingelman–Schlein-style approach to the diffractive event structure [14]. In particular the first part does not agree well with LHC data, and so needs an overhaul.

For the total and elastic cross sections we have chosen to implement two different parametrizations, the parametrization from the COMPAS group as found in the Review of Particle Physics 2016 [15] and a model developed by Appleby and collaborators (ABMST) [16]. In addition to a better fit to the integrated cross sections, these also include a more detailed description of the differential elastic cross sections.

The ABMST model also addresses single diffraction. It is in an ambitious diagrammatical approach, supplemented with a careful description of the resonance shape in the low-mass region, based on comparisons with low-energy data. Unfortunately, as is common in such ansätze, the diffractive cross section asymptotically grows faster with energy than the total one, making it marginally acceptable already at LHC energies and definitely unacceptable for FCC ones. We therefore study possible modifications that would give a more reasonable energy behaviour. Further, while ABMST does not address double or central diffraction, we use the framework of the model to extend it also to these event classes, and in the process need to make further adjustments. Results for the ABMST-based modelling implemented in Pythia are compared with the already existing default framework of Schuler-Sjöstrand (SaS) and Donnachie–Landshoff (DL) [13, 17], and confronted with LHC data.

Furthermore we study the sensitivity to CR by comparing with the Christiansen–Skands QCD-based CR model (CSCR) [18]. This model has no protection against “accidental” rapidity gaps in nondiffractive events, unlike the default CR framework. But it is also not intended to describe (the bulk of) diffraction, and therefore it requires a retuning to provide a sensible combined description. It therefore offers an interesting case study for a tuning task that is likely to become more common in the future.

The plan of the article is as follows: in Sect. 2 we begin by summarising the current status of Pythia 8, the default cross section parametrizations along with the hadronic event properties of diffractive events. In Sect. 3 we describe the new models for total and elastic (differential) cross sections. In Sects. 4, 5 and 6 we extend these to single-, double- and central diffractive (differential) cross sections, respectively. In Sect. 7 we provide some comparisons to LHC data and provide new tunes of the default Pythia 8 model. We end with Sect. 8, where we summarise and provide an outlook to further studies.

## The current status of Pythia 8

Pythia 8 is a multi-purpose event generator aimed at the generation of high-energy events. This includes collisions both of a perturbative and a non-perturbative character, each of which gives contributions to the total collision cross section. In perturbative collisions, the description begins with the matrix element of the hard scattering process in combination with parton distribution functions. This core is dressed up with several other elements such as multiparton interactions, parton showers and hadronisation. In non-perturbative scattering collisions, on the other hand, no standard formulation exists for the core process, and phenomenological models are needed. After the model-dependent choices of the key kinematical variables have been made, the event generation may be continued in a similar manner as for perturbative events, where relevant.

In this paper we focus on the non-perturbative scattering processes, and the generation of these. To set the stage for further improvements, the purpose of this section is to describe the current status of the event generator. This we have split into two parts, beginning with the description of the default cross section models, the SaS/DL one, and then go on to describe the event property aspects that are the same regardless of the choice of model.

### Differential cross sections

In the current version of Pythia 8, the predictions for the total, elastic and diffractive cross sections do not agree so well with measurements performed at the LHC. The current implementation is the parametrization of DL [17] for the total cross section,

1 $$\begin{aligned} \sigma _{\mathrm {tot}}(s)= & {} X^{AB}s^{\epsilon } + Y^{AB}s^{-\eta }, \end{aligned}$$

with $s=E_{\mathrm {CM}}^2$, $\epsilon =0.0808$, $\eta =0.4525$. *A* and *B* denote the initial-state particles, and $X^{AB},\,Y^{AB}$ are specific to each such state. The elastic and diffractive cross sections are described using the parametrization of SaS [13],

2 $$\begin{aligned} \frac{{\mathrm d}\sigma _{\mathrm {el}}}{{\mathrm d}t}= & {} (1+\rho ^2) \, \frac{\sigma _{\mathrm {tot}}^2(s)}{16\pi } \, \exp (B_{\mathrm {el}}(s) \, t) ~, \end{aligned}$$

3 $$\begin{aligned} \frac{{\mathrm d}\sigma _{XB}(s)}{{\mathrm d}t \, {\mathrm d}M_X^2}= & {} \frac{g_{3\mathbb {P}}}{16\pi } \, \frac{\beta _{A\mathbb {P}}(s) \, \beta _{B\mathbb {P}}^2(s)}{M_X^2} \, \exp (B_{XB}(s) \, t) \nonumber \\&\times F_{\mathrm {SD}}(M_X^2,s) ~, \end{aligned}$$

4 $$\begin{aligned} \frac{{\mathrm d}\sigma _{AX}(s)}{{\mathrm d}t \, {\mathrm d}M_X^2}= & {} \frac{g_{3\mathbb {P}}}{16\pi } \, \frac{\beta _{A\mathbb {P}}^2(s) \, \beta _{B\mathbb {P}}(s)}{M_X^2} \, \exp (B_{AX}(s) \, t) \nonumber \\&\times F_{\mathrm {SD}}(M_X^2,s) ~, \end{aligned}$$

5 $$\begin{aligned} \frac{{\mathrm d}\sigma _{XY}(s)}{{\mathrm d}t \, {\mathrm d}M_X^2 \, {\mathrm d}M_Y^2}= & {} \frac{g_{3\mathbb {P}}^2}{16\pi } \, \frac{\beta _{A\mathbb {P}}(s) \, \beta _{B\mathbb {P}}(s)}{M_X^2 \, M_Y^2} \, \exp (B_{XY}(s) \, t) \nonumber \\&\times F_{\mathrm {DD}}(M_X^2,M_Y^2,s) ~, \end{aligned}$$

where indices *X* and *Y* here represent diffractive systems [not to be confused with the coefficients of Eq. (1)], $\rho$ is the ratio of real to imaginary parts of the elastic scattering amplitude at $t=0$ , $\beta _{A\mathbb {P}}$ and $\beta _{B\mathbb {P}}$ are hadron couplings strengths to the Pomeron, and $g_{3\mathbb {P}}$ the triple-Pomeron vertex strength. The slope parameters are defined as

6 $$\begin{aligned} B_{\mathrm {el}}(s)= & {} 2b_{A} + 2b_B + 4s^{\epsilon } - 4.2,\nonumber \\ B_{XB}(s)= & {} 2b_B + 2\alpha _{\mathbb {P}}'\ln \left( \frac{s}{M_X^2}\right) \nonumber \\ B_{AX}(s)= & {} 2b_A + 2\alpha _{\mathbb {P}}'\ln \left( \frac{s}{M_X^2}\right) \nonumber \\ B_{XY}(s)= & {} 2\alpha _{\mathbb {P}}'\ln \left( e^4+\frac{s \, s_0}{M_X^2 \, M_Y^2}\right) , \end{aligned}$$

where $b_i=2.3$ for $i={\mathrm p},\overline{\mathrm p}$, $\alpha _{\mathbb {P}}'=0.25$ GeV$^{-2}$, $s_0=1/\alpha _{\mathbb {P}}'$, and the term $e^4$ is added by hand in order to avoid $B_{\mathrm {DD}}(s)$ to break down for large values of $M_X^2 \, M_Y^2$. Special care was taken to avoid unphysical high-energy behaviours; e.g. a logarithmic *s* dependence of $B_{\mathrm {el}}$ would have lead to $\sigma _{\mathrm {el}}(s) > \sigma _{\mathrm {tot}}(s)$ for large *s*.

Fudge factors are introduced to dampen large (overlapping) mass systems as well as increasing the low-mass “resonance” region, without describing the resonances individually,

7 $$\begin{aligned} F_{\mathrm {SD}}(M_X^2,s)= & {} \left( 1-\frac{M_X^2}{s}\right) \left( 1 + \frac{c_{\mathrm {res}} \, M_{\mathrm {res}}^2}{M_{\mathrm {res}}^2 + M_X^2}\right) \nonumber \\ F_{\mathrm {DD}}(M_X^2, M_Y^2, s)= & {} \left( 1-\frac{(M_X+M_Y)^2}{s}\right) \nonumber \\&\times \left( \frac{s\,m_{{\mathrm p}}^2}{s\,m_{{\mathrm p}}^2 + M_X^2M_Y^2}\right) \nonumber \\&\times \left( 1 + \frac{c_{\mathrm {res}} \, M_{\mathrm {res}}^2}{M_{\mathrm {res}}^2 + M_X^2}\right) \nonumber \\&\times \left( 1 + \frac{c_{\mathrm {res}} \, M_{\mathrm {res}}^2}{M_{\mathrm {res}}^2 + M_Y^2}\right) \end{aligned}$$

where $c_{\mathrm {res}}=2$ and $M_{\mathrm {res}}=2$ GeV for ${\mathrm p}{\mathrm p}$ and ${\mathrm p}\overline{\mathrm p}$.

Central diffraction has been added to Pythia 8, but is not widely used in the experimental communities, hence have not been maintained properly after its inclusion. It is off by default, and is not included in any of the tunes performed by the Pythia 8 collaboration or the experimental communities. Thus the results obtained with it included should not be trusted too far. The cross section is

8 $$\begin{aligned} \sigma _{\mathrm {CD}}(s)= & {} \sigma _{\mathrm {CD}}^{\mathrm {ref}} \frac{\ln ^{1.5}\left( \frac{0.06s}{s_{\mathrm {min}}}\right) }{\ln ^{1.5}\left( \frac{0.06s_{\mathrm {ref}}}{s_{\mathrm {min}}}\right) } ~, \end{aligned}$$

with $\sigma _{\mathrm {CD}}^{\mathrm {ref}}=1.5$ mb, $s_{\mathrm {ref}}=4$ TeV$^2$ and $s_{\mathrm {min}}=1$ GeV$^2$. The diffractive mass is chosen from a $(1 - \xi _1) ({\mathrm d}\xi _1/\xi _1) (1 - \xi _2) ({\mathrm d}\xi _2 / \xi _2)$ distribution, with $\xi _{1,2}$ being the momentum fraction taken from the respective incoming hadron, such that $M_X^2 = \xi _1 \xi _2 s$. The two *t* values are selected according to exponentials with slope $2b_A + \alpha _{\mathbb {P}}'\ln (1/\xi _1)$ and $2b_B + \alpha _{\mathbb {P}}'\ln (1/\xi _2)$, respectively.

The expressions in Eqs. (3)–(5) can be integrated to give the total elastic and diffractive cross sections. This worked reasonably well up to Tevatron energies, but it overshot diffractive cross sections observed at the LHC [19]. Simple overall modification factors were therefore introduced [20] to dampen the growth of the diffractive cross sections [including the CD one in Eq. (8)],

9 $$\begin{aligned} \sigma _i^{\mathrm {mod}}(s)= & {} \frac{\sigma _i^{\mathrm {old}}(s) \, \sigma _i^{\mathrm {max}}}{\sigma _i^{\mathrm {old}}(s) + \sigma _i^{\mathrm {max}}} ~, \end{aligned}$$

where the $\sigma _i^{\mathrm {max}}$ are free parameters. The ansatz allows phenomenology at lower energies to be preserved while giving some reasonable freedom for LHC tunes. It gives asymptotically constant diffractive cross sections, but typically with asymptotia so far away that it is not an issue for current studies.

The kinematical limits for *t* are determined by all the masses in the system. We define the scaled variables $\mu _1=m_A^2/s,\,\mu _2=m_B^2/s,\,\mu _3=M_X^2/s,\,\mu _4=M_Y^2/s$ where $M_X=m_A$ if *A* scatters elastically and $M_Y=m_B$ if *B* scatters elastically. Thus the combinations

10 $$\begin{aligned} C_1= & {} 1 - (\mu _1 + \mu _2 + \mu _3 + \mu _4) + (\mu _1-\mu _2)(\mu _3-\mu _4)\nonumber \\ C_2= & {} \sqrt{(1-\mu _1-\mu _2)^2-4\mu _1\mu _2}\nonumber \\&\times \sqrt{(1-\mu _3-\mu _4)^2-4\mu _3\mu _4}\nonumber \\ C_3= & {} (\mu _3-\mu _1)(\mu _4-\mu _2) \nonumber \\&+ (\mu _1+\mu _4-\mu _2-\mu _3)(\mu _1\mu _4-\mu _2\mu _3), \end{aligned}$$

will lead to the kinematical limits $t_{\mathrm {min}}<t<t_{\mathrm {max}}$.

11 $$\begin{aligned} t_{\mathrm {min}}= & {} -\frac{s}{2}(C_1+C_2)\nonumber \\ t_{\mathrm {max}}= & {} \frac{s^2C_3}{t_{\mathrm {min}}}. \end{aligned}$$

These expressions are directly applicable for elastic scattering and for single and double diffraction. For central diffraction $AB \rightarrow AXB$ they can be applied twice, with $\mu _4 = M_{XB}^2 / s$ for $t_1$ and $\mu _3 = M_{AX}^2 / s$ for $t_2$.

An electromagnetic Coulomb term can be added to describe low-|*t*| elastic scattering. The implementation is here based on the formalism as outlined e.g. in [21, 22]. Introducing an electromagnetic low-|*t*| form factor as

12 $$\begin{aligned} G(t) \approx&\frac{\lambda ^2}{(\lambda - t)^2}, \quad \lambda \approx 0.71~\mathrm {GeV}^2~, \end{aligned}$$

and a Coulomb term phase factor approximation [23, 24]

13 $$\begin{aligned} \phi (t)\approx & {} \pm \alpha _{\mathrm {em}}\left( - \gamma _{\mathrm {E}} - \log \left( - \frac{B_{\mathrm {el}}(s) \, t}{2} \right) \right) ~, \end{aligned}$$

with $\gamma _{\mathrm {E}} \approx 0.577$, $+$ for ${\mathrm p}{\mathrm p}$ and − for ${\mathrm p}\overline{\mathrm p}$, Coulomb and interference terms are added to the hadronic ${\mathrm d}\sigma _{\mathrm {el}}/{\mathrm d}t$ above

14 $$\begin{aligned} \frac{{\mathrm d}\sigma _{\mathrm {el}}^{\mathrm {C+int}}}{{\mathrm d}t}= & {} \frac{4\pi \alpha _{\mathrm {em}}^2\,G^4(t)}{t^2} \pm \frac{\alpha _{\mathrm {em}}\,G^2(t)}{t}\,\nonumber \\&\times \left( \rho \, \cos \phi (t) + \sin \phi (t) \right) \, \sigma _{\mathrm {tot}}(s) \, \nonumber \\&\times \exp \left( \frac{B_{\mathrm {el}}(s) \, t}{2}\right) ~. \end{aligned}$$

The same expression can also be added to the Minimum Bias Rockefeller (MBR) model [25] (and a flexible “set your own” one), while the ABMST and RPP formalisms each introduce the Coulomb corrections as one extra amplitude term, with the full phase expressions of [24]. Numerically the three implementations give very similar results.

### Hadronic event properties

To model a diffractive system, it is convenient to view its internal structure as a consequence of the interaction between two hadronlike objects, e.g. as a $\mathbb {P}B$ subcollision for the $AB \rightarrow AX$ process, in the same spirit as a high-energy nondiffractive ${\mathrm p}{\mathrm p}$ event, where perturbative processes largely shape its structure. Such an approach is not viable for low-mass diffractive systems, however. Therefore the diffractive event generation is split into two regimes, a high-mass and a low-mass one, with a smooth transition between the two. The probability for applying the high-mass description is given by [14]

15 $$\begin{aligned} P_{\mathrm {pert}}= & {} 1 - \exp \left( - \frac{\max (0, M_X - m_{\mathrm {min}})}{m_{\mathrm {width}}}\right) , \end{aligned}$$

with $m_{\mathrm {min}}$ and $m_{\mathrm {width}}$ free parameters, both by default 10 GeV. Note how $P_{\mathrm {pert}}$ vanishes when below $m_{\mathrm {min}}$.

For very low masses, $M_X \le m_B + 1~$GeV for a $\mathbb {P}B$ subcollision, the diffractive system is allowed to decay isotropically into a two-hadron state. Above this limit, but still in the nonperturbative regime, the collision process is viewed as the $\mathbb {P}$ kicking out either a valence quark or a gluon from the incoming hadron *B*. The relative rate of the two is is mass-dependent,

16 $$\begin{aligned} \frac{P(q)}{P(g)}= & {} \frac{N}{M_X^p}, \end{aligned}$$

with *N* and *p* as free parameters, and $M_X$ in GeV. In the former case a single string will be stretched between the kicked-out quark and the left-behind diquark, whereas the latter gives a “hairpin” string topology, going from one remnant valence quark via the struck gluon and back to the remnant diquark. These strings are then allowed to fragment using the Lund fragmentation model [26]. The default values $N = 5$ and $p = 1$ ensures that the double-string topology wins out at higher masses, consistent with what the exchange of a single gluon (a.k.a. a cut Pomeron) is expected to give in ${\mathrm p}{\mathrm p}$ collisions.

In the high-mass regime it is assumed that the diffractive cross section factorises into a Pomeron flux, a Pomeron–proton cross section, and a proton form factor. Together these determine the mass $M_X$ of the diffractive system and the squared momentum transfer *t* in the process. Neither the $\mathbb {P}$ flux nor the $\mathbb {P}{\mathrm p}$ cross section are known from first principles; therefore seven similar but somewhat different $\mathbb {P}$ flux options are available in Pythia 8.

The internal structure of the $\mathbb {P}{\mathrm p}$ system is then considered in an Ingelman–Schlein-inspired picture. Thus perturbative processes are allowed, and $\mathbb {P}$ parton distribution functions (PDFs) are introduced like for a hadron. Standard factorization can be assumed, i.e. cross sections are given by hard-scattering matrix elements convoluted with the PDFs of two incoming partons. Furthermore, the full interleaved shower machinery of Pythia 8 is enabled, giving rise both to initial- and final-state showers and to multiparton interactions in the $\mathbb {P}{\mathrm p}$ system. This results in a more complex colour string structure than in the low-mass regime, which can also be subjected to additional colour reconnection, owing to overlap and crosstalk between the multiple subsystems.

The activity in the $\mathbb {P}{\mathrm p}$ system, as represented e.g. by the average charged multiplicity, can be tuned to roughly reproduce that of a non-diffractive ${\mathrm p}{\mathrm p}$ collision of the same mass. This activity is closely related to the average number of MPIs per event, the calculation of which differs between the two systems by a $\mathbb {P}$ vs. a ${\mathrm p}$ PDF in the numerator, and by $\sigma _{\mathbb {P}{\mathrm p}}^{\mathrm {eff}}$ vs. $\sigma _{{\mathrm p}{\mathrm p}}^{\mathrm {nondiffractive}}$ in the denominator. Given a $\mathbb {P}$ PDF, and assuming the same MPI-framework parameters as in ${\mathrm p}{\mathrm p}$, the $\sigma _{\mathbb {P}{\mathrm p}}^{\mathrm {eff}}$ thus becomes the main (mass-dependent) tuning parameter. In reality the two systems can be different, however, so experimental information on diffractive mass and multiplicity distributions can be used to refine the tune. Be aware that a different choice of PDFs is likely to require a different $\sigma _{\mathbb {P}{\mathrm p}}^{\mathrm {eff}}$ value. Ten different $\mathbb {P}$ PDF sets are implemented [27–30], plus a few toy ones for special purposes. Many of these have been fixed by some convention for the $\mathbb {P}$ flux normalization, that in Pythia could be set differently. In principle most of the $\mathbb {P}$ PDFs should only be used with the associated $\mathbb {P}$ flux, as some of the experimentally provided PDFs do not assume a factorisation of the $\mathbb {P}$ flux and PDF. In practise the two can be chosen independently, as this opens up for comparative studies. Similarly, the convention used for the $\mathbb {P}$ flux normalisation is often dependent on the experimental limits and often normalised to unity at seemingly arbitrary phase space points. Other important aspects, such as the momentum sum rule, are also usually neglected in the PDFs provided by experiments, but often needed in phenomenological studies. Hence all $\mathbb {P}$ PDFs are implemented with the option to be rescaled, e.g. in order to approximately impose the momentum sum rule.

In the MPI framework [31] the joint probability distribution for extracting several partons from a Pomeron needs to be defined. This is done in the same spirit as for protons [32]. MPIs are ordered in a sequence of decreasing $p_{\perp }$ scales, and for the hardest interaction the normal PDFs are used. For subsequent ones the *x* value is interpreted as a fraction of the then remaining $\mathbb {P}$ momentum, thereby ensuring that the momentum sum is not violated. Pomerons are assumed to have no valence quarks; thus the $\mathbb {P}$ PDFs initially only contain gluons and a quark–antiquark-symmetric sea. If a quark is kicked out of the beam, however, flavour conservation requires that an explicit “companion” sea antiquark must also be present in the leftover $\mathbb {P}$, and vice versa. Such a companion is introduced as an extra component of the $\mathbb {P}$ PDF, similar to a valence (anti)quark, with normalization to unity (just like the *d* valence in a proton). Overall momentum is preserved by scaling down the gluon and ordinary sea quark distributions to compensate. If the companion is selected for a subsequent MPI, then that “valence” component is removed, and the gluon and sea components of the $\mathbb {P}$ PDF are scaled back up.

Also initial-state radiation (ISR) requires special attention in the MPI framework. ISR is generated starting from the hard interaction and then evolving backwards, to lower scales and larger *x* values [33]. Such ISR branchings are combined with the MPI generation into one interleaved sequence of falling $p_{\perp }$ scales. As above special consideration has to be given to branchings that change the flavour of the incoming parton, and that can either induce or remove a companion (anti)quark.

Similar to a proton [32], the Pomeron will leave behind a remnant after the MPIs and showers have removed momentum and removed or added partonic content. To begin, assume that only one gluon is kicked out of the incoming $\mathbb {P}$. The remnant will then be in a net colour octet state, which means that two colour strings eventually are stretched to the outgoing partons of the hard collision (or to the other beam remnant). The remnant could only consist of gluons and sea ${\mathrm q}\overline{\mathrm q}$ pairs, since the $\mathbb {P}$ has no valence flavour content, so the simplest representation is as a single gluon or a single ${\mathrm q}\overline{\mathrm q}$ pair. From a physical point of view the two options would give very closely the same end result, since the hairpin string via a gluon remnant eventually would break by the production of ${\mathrm q}\overline{\mathrm q}$ pairs. For convenience, the choice is therefore made to represent the remnant as an octet ${\mathrm u}\overline{\mathrm u}$ or ${\mathrm d}\overline{\mathrm d}$ pair with equal probability. In the general case, further unmatched companion quarks are added to represent the full flavour content needed in the remnant. Most MPI initiators are gluons, however, which carry colour that should be compensated in the remnant. This is addressed by attaching the gluon colour lines to the already defined remnants, which implicitly introduces colour correlations between the initiator partons. Such initial-state correlations can be further enhanced by colour reconnections in the final state. The final colour topology decides how strings connect the outgoing partons after the collision, and thereby sets the stage for the hadron production by string fragmentation.

### Hard diffraction

Recently a framework for truly hard diffractive processes have been implemented into Pythia [34]. This allows for diffractive subprocesses to generate e.g. hard jets, electroweak particles and other internal Pythia processes, unlike the soft-to-medium QCD-only processes that were allowed in the framework described above. This framework decides on whether or not a process is diffractive by evaluating the diffractive part of the proton PDF,

17 $$\begin{aligned} f_{i/{\mathrm p}}^{\mathrm {D}}(x, Q^2)= & {} \int _0^1 {\mathrm d}x_{\mathbb {P}} \, \int _0^1 {\mathrm d}x' \, f_{\mathbb {P}/{\mathrm p}}(x_{\mathbb {P}})\, \nonumber \\&\times f_{i/\mathbb {P}}(x', Q^2) \, \delta (x - x_{\mathbb {P}} x') \nonumber \\= & {} \int _x^1 \frac{{\mathrm d}x_{\mathbb {P}}}{x_{\mathbb {P}}} \, f_{\mathbb {P}/{\mathrm p}}(x_{\mathbb {P}}) \, f_{i/\mathbb {P}} \left( \frac{x}{x_{\mathbb {P}}}, Q^2 \right) , \end{aligned}$$

where $f_{\mathbb {P}/{\mathrm p}}(x_{\mathbb {P}}) = \int f_{\mathbb {P}/{\mathrm p}}(x_{\mathbb {P}},t) \, {\mathrm d}t$, as *t* for the most part is not needed. The ratio $f_{i/{\mathrm p}}^{\mathrm {D}}/f_{i/{\mathrm p}}$ defines the tentative probability for diffraction. A full evolution of the ${\mathrm p}{\mathrm p}$ system is then performed and only the fraction of events passing the evolution without any additional MPIs is kept as diffractive. Additional MPIs between the two hadrons give rise to hadronic activity, which could destroy the rapidity gap between the elastically scattered hadron and the interaction subsystem, which is one of the clear experimental signatures of a diffractive event. If the event survives the no-MPI criterion and is classified as diffractive, the partonic sub-collision is assumed to have happened in a $\mathbb {P}{\mathrm p}$ sub-system. The $\mathbb {P}{\mathrm p}$ system is set up and a full evolution is performed in this subsystem, similar to the method described above.

The no-MPI requirement introduces a gap survival probability determined on an event-by-event basis, unlike other methods used in the literature. As MPIs only occur in hadron–hadron collisions, the framework provides a simple explanation of the differences between the diffractive event rates obtained at HERA and Tevatron. Diffractive fractions and survival probabilities obtained with the new framework show good agreement with experiments, while some distributions show less-than-perfect agreement, see [34] for a discussion. The model is currently only available for single diffraction; future work would be to extend this to both double and central diffraction.

## Total and elastic cross sections

The parametrizations of the total and elastic cross sections are related through the optical theorem. The elastic cross section has historically been well described in the framework of Regge theory, with varying complexity based on the number of exchanges included in the model. Up until the LHC era the simple ansatz of DL [17] using only a Pomeron and an effective Reggeon has described the total cross section surprisingly well. With a simple exponential *t* spectrum, the SaS parametrization [13] extended this to the elastic cross section, and here at least the low-*t* data was well described. But with the higher energies probed at the LHC it has become obvious that these simple parametrizations fail. More complex trajectories have to be introduced in order to describe both the rise of the total cross section and the *t* spectrum of the elastic cross section.

We have chosen to implement two additional models in Pythia 8. One, the model from the COMPAS group as presented in the Review of Particle Physics 2016 [15], is of great complexity, using six different single exchanges as well as some combinations of double exchanges, along with the exchange of three gluons, the latter becoming important at high |*t*|. The other, the newly developed ABMST model [16], is somewhat simpler, extending the original DL model to four single trajectories and all possible combinations of double exchanges between these, along with the triple-gluon exchange for high |*t*| values.

Recent TOTEM collaboration data on elastic scattering hint that none of the traditional models describe all aspects of their data. Specifically, TOTEM obtains a decreasing $\rho$ parameter, and observes no structure in the high-|*t*| region (unpublished, but see e.g. [35]). There is an ongoing discussion in both the theoretical and experimental community on how to describe all data simultaneously. None of the models implemented here do that, specifically they do not predict a decreasing $\rho$ value. Further, the ABMST model does not show any sign of structure at high |*t*|, while the COMPAS one does. Models could be extended to include a maximal odderon, similar to the work of Avila et al. [36, 37] (AGN) and Martynov et al. [38] (FMO), which would be able to describe the decrease in $\rho$. At the time of writing the former has not been fitted to the new TOTEM data and the latter has not been extended to $t \ne 0$. Thus, for now, we have chosen not to implement either in Pythia 8, but we show the FMO model in the relevant figures for completeness. Below we will give short descriptions of each of the fully implemented models.

### The COMPAS model

For the Review of Particle Physics 2016 the COMPAS group [15] has fitted a parametrization of the elastic differential cross section to all available ${\mathrm p}{\mathrm p}$ (upper signs) and ${\mathrm p}\overline{\mathrm p}$ (lower signs) data, using a set of 37 free parameters. The cross sections are functions of the nuclear and amplitude, $T_{\pm }$, as well as the Coulomb amplitude, $T_{\pm }^c$,

18 $$\begin{aligned} \sigma _{\mathrm {tot}}(\sqrt{s})= & {} \frac{\mathrm {Im}\left[ T_{\pm }(s,0)\right] }{\sqrt{s(s-4m_p^2)}}\nonumber \\ \frac{{\mathrm d}\sigma _{\mathrm {el}}}{{\mathrm d}t}(\sqrt{s},t)= & {} \frac{| T_{\pm }(s,t) + T_{\pm }^c |^2}{16\pi (\hbar c)^2\,s(s-4m_p^2)}\nonumber \\ \sigma _{\mathrm {el}}(\sqrt{s})= & {} \frac{1}{16\pi (\hbar c)^2\,s(s-4m_p^2)}\nonumber \\&\times \int _{t_{\mathrm {min}}}^{t_{\mathrm {max}}}{\mathrm d}t \, | T_{\pm }(s,t) |^2. \end{aligned}$$

The Coulomb term, $T_{\pm }^c$, and the nuclear term, $T_{\pm }$, are given as

19 $$\begin{aligned} T_{\pm }^c(s,t)= & {} \pm 8\pi \,\alpha _{\mathrm {em}}\,\exp \left( \mp i\,\alpha _{\mathrm {em}}\,\phi _{\pm }^{\mathrm {NC}}(s,t)\right) \nonumber \\&\times \frac{s}{t} \left( 1 - \frac{t}{\varLambda ^2} \right) ^{-4} \nonumber \\ T_{\pm }(s,t)= & {} F_+(\hat{s},t) \pm F_-(\hat{s},t)\nonumber \\ F_+(\hat{s},t)= & {} F_+^H(\hat{s},t) + F_+^P(\hat{s},t) + F_+^{PP}(\hat{s},t) \nonumber \\&+ F_+^R(\hat{s},t) + F_+^{RP}(\hat{s},t) + N_+(\hat{s},t)\nonumber \\ F_-(\hat{s},t)= & {} F_-^{MO}(\hat{s},t) + F_-^O(\hat{s},t) + F_-^{OP}(\hat{s},t) \nonumber \\&+ F_-^R(\hat{s},t) + F_-^{RP}(\hat{s},t) + N_-(\hat{s},t). \end{aligned}$$

with the exact definitions of the different terms given as stated in [15]. It should be noted that earlier versions of the PDG contains misprints in the definitions above as well as in the crossing of even and odd functions, and the current still contains sign errors for the Coulomb term, so these should be used with care.

### The ABMST model

A somewhat simpler scattering model was proposed by Appleby et al. describing ${\mathrm p}{\mathrm p}$ and ${\mathrm p}\overline{\mathrm p}$ data from ISR to Tevatron energies [16]. The model is based on work by Donnachie and Landshoff [39, 40] describing both elastic scattering and single diffractive scattering, but includes new and more sophisticated fits compared to the ones from Donnachie and Landshoff. In this section the details on the elastic scattering will be given, while the single diffractive scatterings are presented in Sect. 4.

The ABMST model includes both the Coulomb and nuclear amplitudes, as well as the interference between the two. The cross sections are given as

20 $$\begin{aligned} \frac{{\mathrm d}\sigma _{\mathrm {el}}}{{\mathrm d}t}= & {} \pi \, | f_c(s,t)e^{i\alpha \phi (t)} + f_n(s,t) |^2\nonumber \\ \sigma _{\mathrm {el}}(s)= & {} \pi \int _{t_{\mathrm {min}}}^{t_{\mathrm {max}}} {\mathrm d}t \, |f_n(s,t)|^2\nonumber \\ \sigma _{\mathrm {tot}}(s)= & {} \mathrm {Im}\left[ f_n(s,0)\right] , \end{aligned}$$

where the triple-gluon amplitude is left out of the nuclear amplitude [39] when evaluating the total cross section. The Coulomb amplitude from [41] is used and the nuclear amplitude consists of five terms: a hard Pomeron ($\mathbb {P}_h$), a soft Pomeron ($\mathbb {P}_s$), the $f_2,a_2$ Regge trajectory ($\mathbb {R}_1$), the $\rho ,\omega$ Regge trajectory ($\mathbb {R}_2$) and a triple-gluon exchange amplitude,

21 $$\begin{aligned} f_n(s,t)= & {} A_{ggg}(t) + \sum _{i=\mathbb {P}_h, \mathbb {P}_s, \mathbb {R}_1, \mathbb {R}_2} A_i(s,t). \end{aligned}$$

Also included is a double exchange term, where e.g. two Pomerons are exchanged. Exact definitions of the various terms are found in [16, 42]. It should be noted that that the cross sections are only valid down to $\sqrt{s}=10$ GeV, and that the fits have only been performed up to UA1 energies. We thus expect good agreement in this energy range, whereas the fit might disagree with data outside of it.

### The FMO model

The FMO model [38] includes the maximal odderon, excluded by hand in the COMPAS model. The odderon has been a controversial subject ever since its introduction, and so far no signs of it has been observed. The main feature of its introduction is that the difference between ${\mathrm p}{\mathrm p}$ and $\overline{\mathrm p}{\mathrm p}$ total cross sections is not vanishing at high energies. Similarly the $\rho$ values will deviate at high energies. The FMO model only includes the $t=0$ contribution and can be written as

22 $$\begin{aligned} \sigma _{\mathrm {tot}}(s)= & {} \frac{\mathrm {Im}T_{\pm }(s,0)}{\sqrt{s(s - 4 m_{{\mathrm p}}^2}}\nonumber \\ T_{\pm }= & {} F_+^H \pm F_-^{MO} + F_+^R \pm F_-^R, \end{aligned}$$

with the exact definitions of the crossing-odd and -even amplitudes found in [38].

### Comparisons with data

**Fig. 2:**
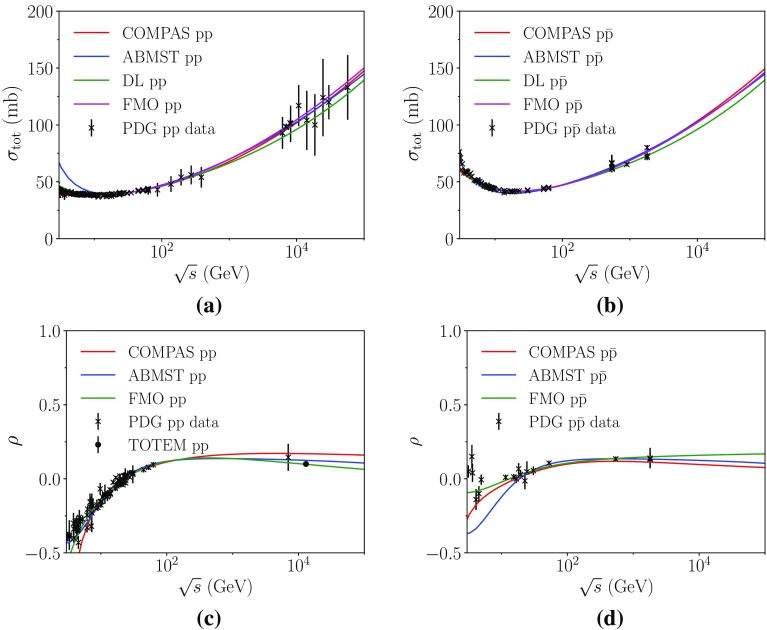
The total cross section parametrizations in **a** ${\mathrm p}{\mathrm p}$ and **b** ${\mathrm p}\overline{\mathrm p}$ processes. The ratio of real to imaginary parts of the elastic amplitude at $t=0$ for ${\mathrm p}{\mathrm p}$ (**c**) and ${\mathrm p}\overline{\mathrm p}$ (**d**). Note that the SaS model has been left out in **c**, **d**, as $\rho$ is a constant here, that can be set freely by the user. Data from PDG [15]

In Fig. 2a, b we show the above parametrizations of the total cross section and in Fig. 2c, d the $\rho$ parameter, for ${\mathrm p}{\mathrm p}$ and ${\mathrm p}\overline{\mathrm p}$ processes respectively. Note how the ABMST $\sigma _{\mathrm {tot}}^{{\mathrm p}{\mathrm p}}$ parametrization rises at $\sqrt{s}<10$ GeV, a consequence of it not being fitted to this range. We do not aim to describe so low energies in Pythia 8, so this is not an issue. Both the ABMST and COMPAS parametrizations well describe the LHC data points in ${\mathrm p}{\mathrm p}$, and seem to favour the higher of the Tevatron data points in ${\mathrm p}\overline{\mathrm p}$ processes, unlike the original DL parametrization available in Pythia 8. In Fig. 2c the $\rho$ is well described by all three parametrizations, below LHC energies. But at LHC the latest TOTEM value [36] is described only by the FMO model, which explicitly includes the maximal odderon term in order for $\rho$ to decrease here. This term also gives rise to the difference in $\rho$ for ${\mathrm p}{\mathrm p}$ and ${\mathrm p}\overline{\mathrm p}$ processes, as seen in Fig. 2c, d, a difference not present in the other two models.

**Fig. 3:**
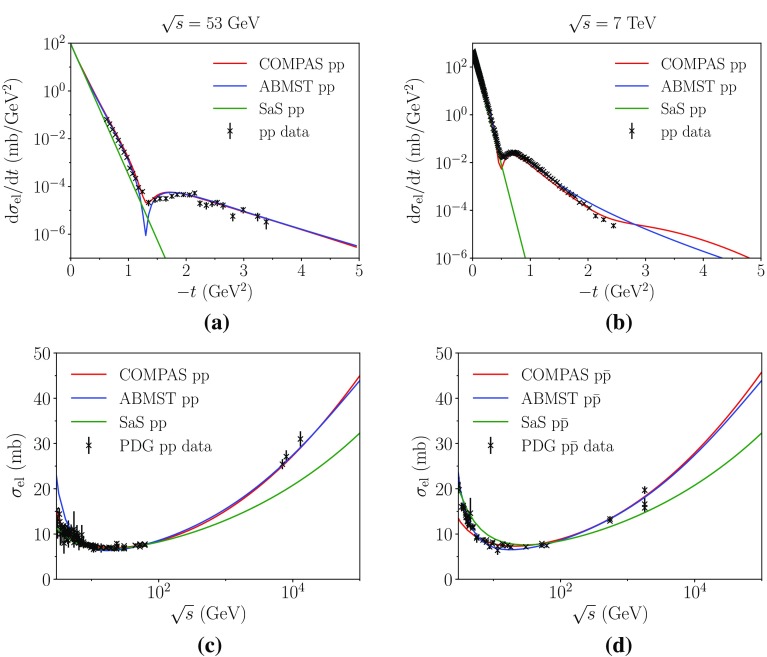
The elastic differential cross section parametrizations in ${\mathrm p}{\mathrm p}$ collisions at 53 GeV (**a**) and 7 TeV (**b**). The integrated elastic cross section parametrizations in **c** ${\mathrm p}{\mathrm p}$ and **d** ${\mathrm p}\overline{\mathrm p}$ processes. Data from PDG [15]

In Fig. 3 we show the available parametrizations of the elastic differential (a, b) and integrated (c, d) cross sections for ${\mathrm p}{\mathrm p}$ and ${\mathrm p}\overline{\mathrm p}$ processes. Here it is evident that the pure exponential description used by SaS only makes sense for small |*t*|. Both the COMPAS and ABMST parametrizations have been fitted to the $\sqrt{s}=23$ GeV data, but not to the 7 TeV data. Here it seems that the COMPAS parametrization prefers a larger dip than seen in data, while it captures the high-|*t*| region slightly better than the ABMST parametrization. It is also evident that SaS underestimates the rise of the total elastic cross section, whereas the other two do quite well.

## Single diffractive cross sections

As we proceed to the topologies of diffraction, the situation is more complicated than for total and elastic cross sections. The experimental definition of diffraction is based on the presence of rapidity gaps, but such gaps are subject to random fluctuations in the hadronization process, and therefore cannot be mapped one-to-one to an underlying colour-singlet-exchange mechanism. Also the separation between single, double and central diffraction is not always so clearcut. Some single-diffractive data is available at lower energies, but much of it is old and of varied quality. This will of course affect any model trying to describe these topologies, as usually there are model parameters that have to be fitted to data. To the best of our knowledge, only a few models actually try to fit data fully differentially in both *s*, $M_X^2$ and *t*. The normal ansatz is instead to define an *s*-independent $\mathbb {P}$ flux, with factorized $\xi$ and *t* distributions, e.g. of the form $({\mathrm d}\xi / \xi ^{1+ \delta }) \, \exp (b \, t) \, {\mathrm d}t$ [27, 43–45] where $\delta$ is a small number. The t-integrated $\xi$ distribution is then directly mapped on to an $M_X^2 = \xi s$ spectrum.

The COMPAS group has not made any attempts to describe other topologies than the elastic, neither has the FMO model. Hence, in addition to the already implemented SaS and MBR models, we are left with the ABMST model as a new alternative, that gives a full description of the single diffractive topologies. This model has been fitted to differential data in the energy range $17.2< \sqrt{s} < 546$ GeV and in the *t* range $0.015< |t| < 4.15$ GeV$^2$, and is thus expected to give a reasonable prediction in this range. The model, however, has some unfortunate features, which we will discuss in a later section. But first an introduction to the basics of the model itself.

### The ABMST model

In [16] the authors present a model for single diffractive dissociation inspired by Donnachie and Landshoff. They operate in two regimes, high and low mass diffraction, separated at

23 $$\begin{aligned} M_{\mathrm {cut}}(s)= & {} {\left\{ \begin{array}{ll} 3 &{} s < 4000\,\mathrm {GeV}^2\\ 3 + 0.6\ln \left( \frac{s}{4000}\right) &{} s > 4000\,\mathrm {GeV}^2\\ \end{array}\right. }. \end{aligned}$$

In the high mass regime, they use a triple-Regge model with two components; An effective Pomeron and a degenerate Reggeon term. In order for the unknown phases of the propagators to vanish, they require that the two *t*-dependent propagators in the diagrams contributing to the single diffractive cross section are equal. This results in four diagrams; $\mathbb {P}\mathbb {P}\mathbb {P}$, $\mathbb {P}\mathbb {P}\mathbb {R}$, $\mathbb {R}\mathbb {R}\mathbb {P}$, $\mathbb {R}\mathbb {R}\mathbb {R}$. The authors also include pion exchange in the differential cross section arriving at

24 $$\begin{aligned} \frac{{\mathrm d}^2\sigma _{\mathrm {HM}}}{{\mathrm d}t{\mathrm d}\xi }(\xi , s,t)= & {} f_{\mathbb {P}\mathbb {P}\mathbb {P}}(t)\xi ^{\alpha _{\mathbb {P}}(0)-2\alpha _{\mathbb {P}}(t)} \left( \frac{s}{s_0}\right) ^{\alpha _{\mathbb {P}}(0)-1}\nonumber \\&+ f_{\mathbb {P}\mathbb {P}\mathbb {R}}(t)\xi ^{\alpha _{\mathbb {R}}(0)-2\alpha _{\mathbb {P}}(t)} \left( \frac{s}{s_0}\right) ^{\alpha _{\mathbb {R}}(0)-1}\nonumber \\&+ f_{\mathbb {R}\mathbb {R}\mathbb {P}}(t)\xi ^{\alpha _{\mathbb {P}}(0)-2\alpha _{\mathbb {R}}(t)} \left( \frac{s}{s_0}\right) ^{\alpha _{\mathbb {P}}(0)-1}\nonumber \\&+ f_{\mathbb {R}\mathbb {R}\mathbb {R}}(t)\xi ^{\alpha _{\mathbb {R}}(0)-2\alpha _{\mathbb {R}}(t)} \left( \frac{s}{s_0}\right) ^{\alpha _{\mathbb {R}}(0)-1}\nonumber \\&+ \frac{g_{\pi \pi {\mathrm p}}^2}{16\pi ^2}\frac{|t|}{(t-m_{\pi }^2)^2} F^2(t)\xi ^{1-2\alpha _{\pi }(t)}\nonumber \\&\times \sigma _{\pi ^0{\mathrm p}}(s\xi ), \end{aligned}$$

with trajectories and parameter choices found in [16]. Each of the effective three-Reggeon couplings are given as

25 $$\begin{aligned} f_{kki}(t)= & {} A_{kki}e^{B_{kki}t}+C_{kki}, \end{aligned}$$

except for the triple-Pomeron coupling, which is modified as

26 $$\begin{aligned} f_{\mathbb {P}\mathbb {P}\mathbb {P}}(t)= & {} 0.4 + 0.5t ~~~ \mathrm {for}~~~ -0.25 \le t< -10^{-4}\nonumber \\ f_{\mathbb {P}\mathbb {P}\mathbb {P}}(t)= & {} (A_{\mathbb {P}\mathbb {P}\mathbb {P}}e^{B_{\mathbb {P}\mathbb {P}\mathbb {P}}t}+C_{\mathbb {P}\mathbb {P}\mathbb {P}}) \left( \frac{t}{t-0.05}\right) \nonumber \\&~~~~~\mathrm {for} ~~~ -1.15 \le t< -0.25\nonumber \\ f_{\mathbb {P}\mathbb {P}\mathbb {P}}(t)= & {} (A_{\mathbb {P}\mathbb {P}\mathbb {P}}e^{B_{\mathbb {P}\mathbb {P}\mathbb {P}}t}+C_{\mathbb {P}\mathbb {P}\mathbb {P}}) \left( \frac{t}{t-0.05}\right) \nonumber \\&\times [1 + 0.4597(|t|-1.15) + 5.7575(|t|-1.15)^2] \nonumber \\&~~~~~ \mathrm {for} ~~~ -4 \le t < 1.15 . \end{aligned}$$

Four resonances are modelled in the low-mass regime, along with a background from the high-mass regime and a contact term matching the two regimes smoothly. The resonances are excited states of the proton, each a unit of angular momentum higher than the previous one. The resonances are parametrized by Breit–Wigner shapes with masses $m_i$, widths $\varGamma _i$ and couplings $c_i$,

27 $$\begin{aligned} \frac{{\mathrm d}^2\sigma _{\mathrm {res}}}{{\mathrm d}t{\mathrm d}\xi }(\xi ,s,t)= & {} \frac{e^{13.5(t+0.05)}}{\xi } \nonumber \\&\times \sum _{i=1}^4\left[ \frac{c_im_i\varGamma _i}{(\xi s - m_i^2)^2 + (m_i\varGamma _i)^2}\right] ~, \end{aligned}$$

with exact definitions found in the paper. The background is assumed quadratic and vanishes at a threshold, $\xi _{\mathrm {th}} = \frac{(m_{{\mathrm p}} + m_{\pi })^2}{s}$,

28 $$\begin{aligned} A_{\mathrm {bkg}}(\xi ,s,t)= & {} a(s,t)(\xi - \xi _{\mathrm {th}})^2 + b(s,t)(\xi -\xi _{\mathrm {th}}). \end{aligned}$$

A matching term between the high- and low-mass regions is subtracted from the resonances to avoid any discontinuities at $\xi _{\mathrm {cut}}$, and parametrized such that it is equal to the magnitude of the resonance term at the matching point.

### Comments on the ABMST model

**Fig. 4:**
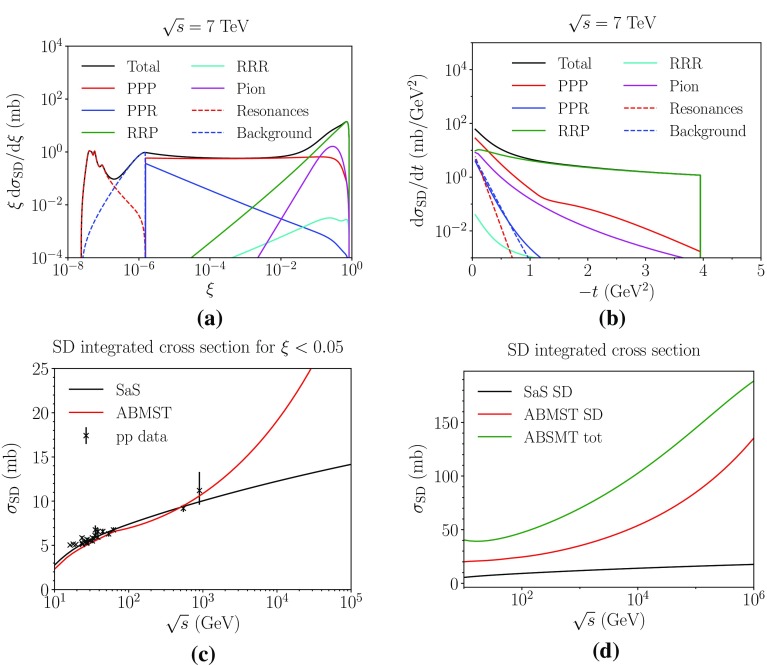
The different components of the ABMST model for single diffraction as a function of **a** $\xi$ and **b** *t* at 7 TeV. The integrated single diffractive cross section as a function of $\sqrt{s}$ for $\xi <0.05$ (**c**) and in the full single diffractive phase space (**d**). Data from references in [16]

In Fig. 4a, b we show the different components of the ABMST model at an energy of $\sqrt{s}=7$ TeV along with the integrated cross sections in Fig. 4c, d. We have several comments to these distributions, as they show some unexpected features.

To begin, consider the differential distribution in Fig. 4a. Here the cross section (multiplied by a factor of $\xi$ for visibility) is shown as a function of $\xi$, displaying both the low-mass resonances and the high-mass Regge terms. Note, however, the dip between these two regimes, a decrease of a factor of 10. This is a feature of the background modelling, whereas one would expect a more smooth transition between the two regimes. There is no physical motivation as to why the Regge trajectories should have a quadratic behaviour at low masses, since none of the terms show this behaviour at higher masses. One could imagine a simple continuation of the high-mass background to lower masses, with the resonances added on top. But this would likely cause too high a cross section in the low-mass region, hence requiring a remodelling of the background description to avoid too high a low-mass cross section.

Similarly unexpected is the increase of the cross section at higher masses ($\xi \sim 1$), induced by the triple-Reggeon and pion terms. The larger the mass of the system the smaller the rapidity gap between the diffractive system and the elastically scattered proton. The rule of thumb is that $\varDelta y_{\mathrm {gap}} \approx \ln (\xi )$, so for large $\xi$ there will essentially be no gap at all. The diffractive system will simply look like a non-diffractive one, making it impossible to distinguish between the two experimentally. The rise at $\xi \sim 1$ also introduces a vast increase with energy in the integrated cross section, making the single-diffractive cross section dominate at large energies, which leaves little room for other processes, see Fig. 4d. The authors themselves have tried to dampen the increase of the cross section by allowing the mass cut, separating the low- and high-mass regimes, to vary with *s*, Eq. (23). Unfortunately the introduced dampening gives rise to a kink in the integrated cross section where the dampening kicks in, at $\sqrt{s}\sim 60$ GeV, and does not dampen the cross section sufficiently at high energies.

In Fig. 4b we show the ABMST model differential in *t*. Noteworthy are the *t*-independent terms $C_{kki}$ and the sharp cutoff at $t=-\,4$ GeV$^2$, both of which are unphysical on their own. That is, if the sharp cutoff is disregarded, then all but the pion and triple-Pomeron terms become constant at large |*t*|, lacking any form factor suppression for scattering a proton without breaking it up. The choice of *t* parametrization shape was based on the goodness-of-fit, and not on any physical grounds. The authors note that the parametrization as such gives too large a cross section at high energies, hence the modification of the Pomeron coupling, as this dominates at high energies. The *t* ansatz may also cause problems if used in other diagrams, e.g. in the extension to double and central diffraction that we will introduce later.

As Pythia 8 aims to describe current and future colliders, the need for a more sensible high-energy behaviour of the ABMST model is evident. It is not realistic to have a model where single diffraction and elastic scattering almost saturates the total cross section at FCC energies (at $10^5$ GeV $\sigma _{\mathrm {tot}} - \sigma _{\mathrm {el}} - \sigma _{\mathrm {SD}} \approx 145 - 45 - 80 \approx 20$ mb). At the same time we want to make use of the effort already put into the careful tuning to low-energy and low-diffractive-mass data. We have thus chosen to provide a modified version of the ABMST model, addressing the problems discussed above, as described in the next section, while retaining the good aspects of the ABMST model. Both the modified and the original version of the ABMST model are made available in the latest Pythia 8 release.

### The modified ABMST model

To smoothen the dip between the low-mass and high-mass regions, several background terms have been studied, such as a linear background becoming constant at threshold, a combination of the linear and the quadratic background and, as an extreme, a continuation of the high-mass background. The best results was found with the combination of the linear and quadratic,

29 $$\begin{aligned} A_{\mathrm {bkg}}(s)= & {} {\left\{ \begin{array}{ll} A_{\mathrm {bkg}}^{\mathrm {quadratic}} &{} M_X< M_{4}\\ A_{\mathrm {bkg}}^{\mathrm {linear}} &{} M_{4}< M_X < M_{\mathrm {cut}}\\ \end{array}\right. }, \end{aligned}$$

where $M_4$ is the mass of the fourth resonance.

**Fig. 5:**
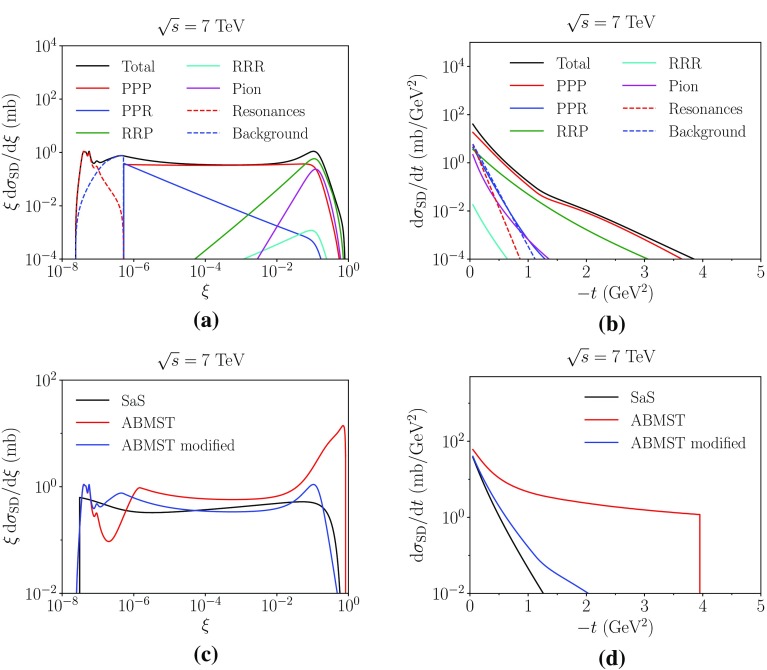
The different components of the modified ABMST model for single diffraction as a function of **a** $\xi$ and **b** *t* at 7 TeV. The same distributions are shown in **c**, **d**, where we compare the two models ABMST and ABMST modified to the SaS model

The new parametrization of the high-mass background in the low-mass region does smoothen the decrease between the two regions, but in itself does increase the integrated cross section. We tame the integrated cross section by introducing a multiplicative rescaling of the high-mass region, as well as a different $M_{\mathrm {cut}}$ parametrization. Again several possibilities have been tried, and best results were obtained for a $\ln ^2(s)$-dependent $M_{\mathrm {cut}}$ and rescaling. That is, $M_{\mathrm {cut}} = 3+c\,\ln ^2(s/s_0)$ GeV and the rescaling factor is $3 / (3+c\,\ln ^2(s/s_0))$, with *c* a free parameter and $s_0 = 100$ GeV$^2$, which is also where the rescaling begins, so as to avoid kinks in the distributions.

While this change reduces the cross section at intermediate $\xi$ values, it does not address the strong rise near $\xi = 1$. This is an unobservable behaviour, as already argued, and therefore we also introduce a dampening factor $1 / (1 + (\xi \exp (y_{\mathrm {min}}))^p)$ for the high-mass region. Here $y_{\mathrm {min}}$ is the gap size where the dampening factor is 1 / 2 and *p* regulates how steeply this factor drops around $y_{\mathrm {min}}$; by default $y_{\mathrm {min}} = 2$ and $p = 5$.

Separately, we wish to remove the artificial cut at $t = -4$ GeV$^2$, in favour of a shape that is valid at all *t* scales. To this end, couplings are modified as

30 $$\begin{aligned} f_{kki}^{\mathrm {ABMST}}(t) \rightarrow f_{kki}^{\mathrm {mod}}(t)= & {} (A_{kki}+C_{kki}^{\mathrm {mod}})e^{B_{kki}^{\mathrm {mod}}t}, \end{aligned}$$

where two new parameters $C_{kki}^{\mathrm {mod}}$ and $B_{kki}^{\mathrm {mod}}$ are introduced. These are fixed by the two requirements that the integral over *t* and the average *t* value should remain unchanged relative to the original ABMST values. Note, however, that we do not modify the $\mathbb {P}\mathbb {P}\mathbb {P}$ part, as this already has the desired decreasing behaviour at high |*t*|. Besides these modifications, a minimum diffractive slope $B_{SD} = 2$ is introduced, to avoid any unphysical situations where the slope could become negative.

In Fig. 5 we show the components of the modified ABMST model as a function of $\xi$ (a) and *t* (b). The improvements of the modifications are clearly seen, as the dip between the low- and high-mass description has decreased, the high-$\xi$ region has been dampened and none of the components become constant at large |*t*|. In Fig. 5c, d the two ABMST models are compared to the SaS model available in Pythia 8 as default. We note that the modified ABMST model shows better agreement with the SaS model at intermediate $\xi$ values, where SaS is in rough agreement with data, while retaining some features of the ABMST model, such as the detailed resonance structure.

**Fig. 6:**
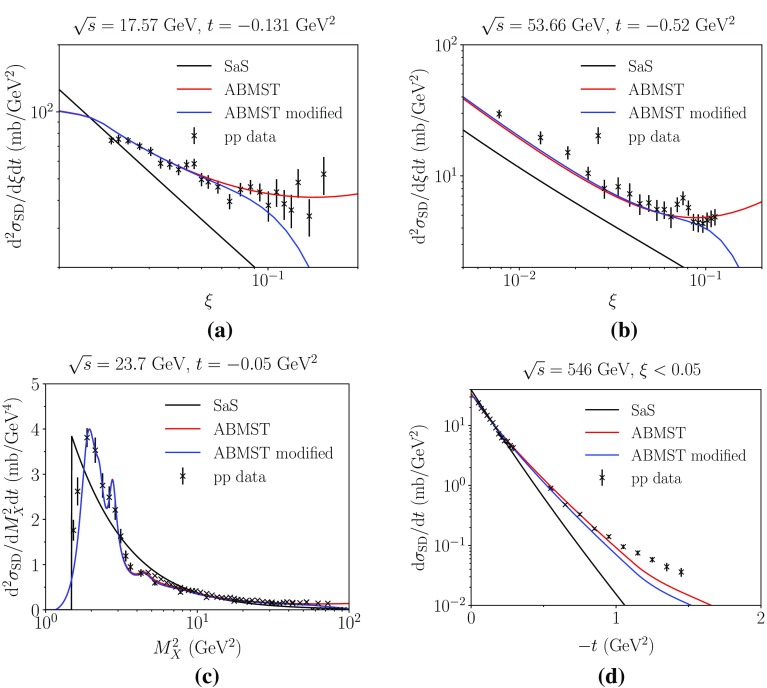
The single diffractive differential cross section parametrizations in ${\mathrm p}{\mathrm p}$ collisions at $\sqrt{s}$ 17.57 GeV with $t=-\,0.131$ GeV$^2$ (**a**) and 53.66 GeV with $t=-\,0.52$ GeV$^2$ (**b**). The mass-spectrum showing the resonances at $\sqrt{s}=$ GeV and $t=-$ GeV$^2$ (c). The integrated *t* spectrum at $\sqrt{s}=$ GeV (d). Data from references in [16]

**Fig. 7:**
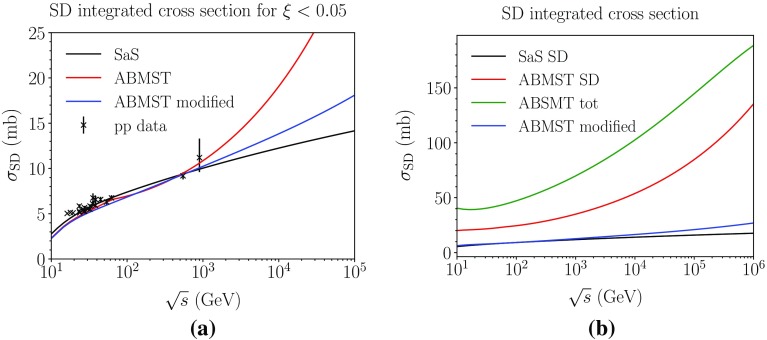
The integrated single diffractive cross section at different energies for $\xi <0.05$ (**a**) and in the full phase space (**b**). Data from references in [16]

In Fig. 6 we show the comparison between the implemented models and the low-energy data used in [16]. It is clear that the SaS model does not agree with data, while both the original and the modified ABMST model describe data reasonably well. In Fig. 7a, b the integrated cross sections of all three models are shown in the restricted (a) and full (b) phase space. The growth of the ABMST model has been tamed by our modifications. Insofar as the SaS model seems to be on the high side relative to data, and the modified ABMST is slightly higher, it may become necessary to finetune further for LHC applications. To this end we have introduced an optional overall scaling factor $k(s/m_p^2)^p$, with *k*, *p* being tuneable parameters.

The bulk of the modifications applied to the ABMST framework are intended to tame the high-energy behaviour of the model. One could have used an eikonal approach to the same end, e.g. in the spirit of [46]. This would require a different set of assumptions, however, such as the impact-parameter shape of the different diffractive topologies, and therefore not be any less arbitrary. For now we therefore stay with the current framework and instead proceed to address other shortcomings of the ABMST model, namely the lack of double and central diffraction.

## Double diffractive cross sections

The ABMST model only provides a description of the single diffractive differential cross section. We can extend this to double diffractive systems, by extracting the vertices and propagators from the single diffractive framework and using them in double diffractive diagrams. Figure 1e shows a double diffractive diagram, where $\mathbb {X}$ is one of the Reggeons used in the single diffractive framework. Thus several diagrams are obtained with Reggeons *i*, *j*, *k* (where *i*, *j* are connected to the proton and *k* are in the loop). Similar as for single diffraction, in order for the unknown phases in the propagators to vanish, the requirement of equal Reggeons is enforced in the loop. The fact that there are two different mass regimes (low and high) for the two diffractive systems *X* and *Y* gives four different combinations.

If both systems have high mass, $M_{X,Y} > M_{\mathrm {cut}}$, the diagram of Fig. 1e implies a cross section

31 $$\begin{aligned} \frac{\mathrm {d}^3\sigma }{\mathrm {d}t\mathrm {d}M_X^2\mathrm {d}M_Y^2}= & {} \sum _{ijk}\frac{g_{i{\mathrm p}}(0)g_i^{kk}(t)g_{j{\mathrm p}}(0)g_j^{kk}(t)}{16\pi M_X^2 M_Y^2} \nonumber \\&\times \left( \frac{M_X^2}{s_0}\right) ^{\alpha _i(0)-1} \left( \frac{M_Y^2}{s_0}\right) ^{\alpha _j(0)-1}\nonumber \\&\times \left( \frac{ss_0}{M_X^2M_Y^2}\right) ^{2\alpha _k(t)-2}. \end{aligned}$$

Changing variables to $\xi =M^2/s$ and collecting the terms one obtains

32 $$\begin{aligned} \frac{\mathrm {d}^3\sigma }{\mathrm {d}t\mathrm {d}\xi _X\mathrm {d}\xi _Y}= & {} \frac{1}{16\pi }\sum _{ijk}\left( \frac{s}{s_0}\right) ^{2 -2\alpha _k(t)}\nonumber \\&\times g_{i{\mathrm p}}(0)g_i^{kk}(t)\xi _X^{\alpha _i(0)-2\alpha _k(t)}\left( \frac{s}{s_0}\right) ^{\alpha _i(0)-1}\nonumber \\&\times g_{j{\mathrm p}}(0)g_j^{kk}(t)\xi _Y^{\alpha _j(0)-2\alpha _k(t)}\left( \frac{s}{s_0}\right) ^{\alpha _j(0)-1}.\nonumber \\ \end{aligned}$$

From the single diffractive framework one has that

33 $$\begin{aligned} \frac{\mathrm {d}^2\sigma _{\mathrm {HM}}}{\mathrm {d}t\mathrm {d}\xi }= & {} \frac{1}{16\pi }\sum _{ik} g_{k{\mathrm p}}^2(t)g_{i{\mathrm p}}(0)g_i^{kk}(t)\nonumber \\&\times \xi ^{\alpha _i(0)-2\alpha _k(t)}\left( \frac{s}{s_0}\right) ^{\alpha _i(0)-1}, \end{aligned}$$

where we can recognise a part of the single diffractive cross section in the double diffractive cross section,

34 $$\begin{aligned} \frac{\mathrm {d}^3\sigma }{\mathrm {d}t\mathrm {d}\xi _X\mathrm {d}\xi _Y}= & {} \frac{1}{16\pi }\sum _{k}\left( \frac{s}{s_0}\right) ^{2 -2\alpha _k(t)}\nonumber \\&\times \left[ \frac{16\pi }{g_{k{\mathrm p}}^2(t)}\frac{\mathrm {d}\sigma _{\mathrm {HM}}}{\mathrm {d}t\mathrm {d}\xi _X}\right] \left[ \frac{16\pi }{g_{k{\mathrm p}}^2(t)}\frac{\mathrm {d}\sigma _{\mathrm {HM}}}{\mathrm {d}t\mathrm {d}\xi _Y}\right] \nonumber \\= & {} \frac{\mathrm {d}\sigma _{\mathrm {HM}}}{\mathrm {d}t\mathrm {d}\xi _X} \frac{\mathrm {d}\sigma _{\mathrm {HM}}}{\mathrm {d}t\mathrm {d}\xi _Y} \sum _{k} \frac{16\pi }{g_{k{\mathrm p}}^4(t)}\left( \frac{s}{s_0}\right) ^{2 - 2\alpha _k(t)}. \end{aligned}$$

A similar diagrammatic method can be used for the low-mass region, so all four $(M_X, M_Y)$ regions can generically be described as

35 $$\begin{aligned} \frac{\mathrm {d}^3\sigma }{\mathrm {d}t\mathrm {d}\xi _X\mathrm {d}\xi _Y}= & {} \frac{\mathrm {d}\sigma _{\mathrm {SD}}}{\mathrm {d}t\mathrm {d}\xi _X} \frac{\mathrm {d}\sigma _{\mathrm {SD}}}{\mathrm {d}t\mathrm {d}\xi _Y} \sum _{k=\mathbb {P},\mathbb {R}} \frac{16\pi }{g_{k{\mathrm p}}^4(t)}\left( \frac{s}{s_0}\right) ^{2 - 2\alpha _k(t)}\nonumber \\\rightarrow & {} \frac{\mathrm {d}\sigma _{\mathrm {SD}}}{\mathrm {d}t\mathrm {d}\xi _X} \frac{\mathrm {d}\sigma _{\mathrm {SD}}}{\mathrm {d}t\mathrm {d}\xi _Y} \frac{16\pi }{g_{\mathbb {P}{\mathrm p}}^4(t)}\left( \frac{s}{s_0}\right) ^{2 - 2\alpha _{\mathbb {P}}(t)}. \end{aligned}$$

In the last step we have taken the high-energy limit, where the Pomeron term dominates. The last term can then be recognised as the inverse of the elastic cross section in the same limit, and hence [4]

36 $$\begin{aligned} \frac{\mathrm {d}^3\sigma _{\mathrm {DD}}}{\mathrm {d}t\mathrm {d}\xi _X\mathrm {d}\xi _Y}\approx & {} \frac{\mathrm {d}^2\sigma _{\mathrm {SD}}}{\mathrm {d}t\mathrm {d}\xi _X} \frac{\mathrm {d}^2\sigma _{\mathrm {SD}}}{\mathrm {d}t\mathrm {d}\xi _Y} \, \Bigg / \, \frac{\mathrm {d}\sigma _{\mathrm {el}}^{\mathbb {P}}}{\mathrm {d}t}. \end{aligned}$$

In principle this formulation holds only at high energies, and only when using the Pomeron as exchanged particle in all parts of the diagram in Fig. 1e. Nevertheless it offers the best way to introduce double diffraction as a natural extension of the ABMST single diffractive machinery, and is the one we will choose.

**Fig. 8:**
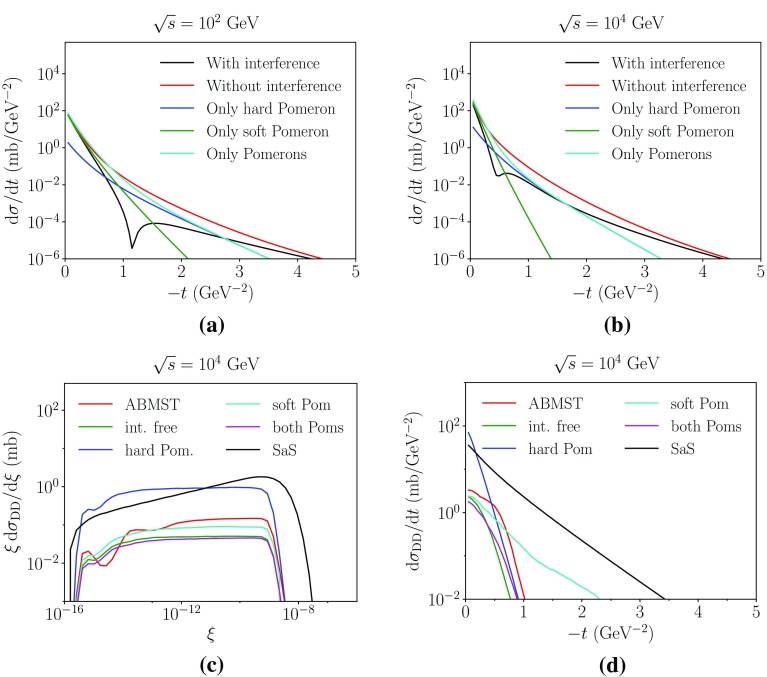
The effect of using only a subset of the available Pomerons in the elastic parametrisation, as used in the expression for the double diffractive cross section, Eq. (36). In **a**, **b** the elastic differential cross section is shown as a function of *t* at two energies. In **c**, **d** the effect of these on the double diffractive distributions are shown as a function of $\xi =\xi _1\xi _2$ and *t*, respectively. Note that the “Pure ABMST” has a minimal slope of $B_{DD}=2$ such as to avoid the dip structure of the elastic description

One of the drawbacks of this approach is that accidental dips in the elastic cross section denominator can come to blow up the double diffractive cross section beyond what reasonably should be expected. Therefore a slightly modified elastic cross section is called for in this context. In Fig. 8a, b the different Pomeron contributions to the elastic differential cross section are shown at two energies, along with the full description and an interference-free description of the form

37 $$\begin{aligned} \frac{{\mathrm d}\sigma _{\mathrm {el}}}{{\mathrm d}t}= & {} \frac{|\sum _iA_i(s,t)|^2}{16\pi } \simeq \frac{\sum _i|A_i(s,t)|^2}{16\pi } \end{aligned}$$

where *i* runs over all four terms. Notice that the hard and soft Pomeron contributions dominate in two different regions. Hence a reasonable approximation would be to use the soft Pomeron term in the low-|*t*| range and the hard Pomeron term in the high-|*t*| range. In practise we use the combination of the hard and soft Pomerons, so as to avoid splitting Eq. (36) into two different *t* ranges.

Figure 8c, d show the effect of the various elastic parametrizations on the double diffractive distributions. Note the normalisation difference between the hard-Pomeron-only description and the others, a difference that arises since the hard Pomeron term is not the dominant one in the low-|*t*| region, where most of the cross section is. On the other hand, the soft-Pomeron-only *t* spectrum is much wider than the other distributions shown, since the soft Pomeron contribution to elastic scattering falls off much steeper with *t*. The “Pure ABMST”, interference-free ABMST and “ABMST both Poms” appear to have the same shapes in Fig. 8c, d. They differ somewhat in normalisation, as is expected given that the two latter correspond to somewhat larger elastic cross sections.

To correct for the possible suppressions arising from the chosen approximation of the elastic cross section, and from the underestimation implied by the step taken in Eq. (35), we introduce a scaling factor similar to the one introduced in the single diffractive framework. A minimal double diffractive slope can also be enforced, such as to avoid any unphysical situations. As a final modification, an option to reduce topologies without a rapidity gap is applied in the region where both of the systems are of very large masses. Again, this is to be able to distinguish the double-diffractive system from the non-diffractive ones.

As two different parametrizations are available in the ABMST framework for single diffraction, several choices for the double diffractive framework exists. Presented here are results with three choices:

- Pure ABMST: the original ABMST single diffractive model together with the elastic cross sections using only Pomerons, with the minimal double diffractive slope and with reduced vanishing-gap topologies.
- Model 1: The modified ABMST model for the single diffractive cross section, with the only-Pomerons elastic cross section. A minimal slope is used and the vanishing-gap topologies are also reduced here.
- Model 2: Model 1 scaled with the tuneable factor $k(s/m_p^2)^p$, where by default $k=2$ and $p=0.1$.

**Fig. 9:**
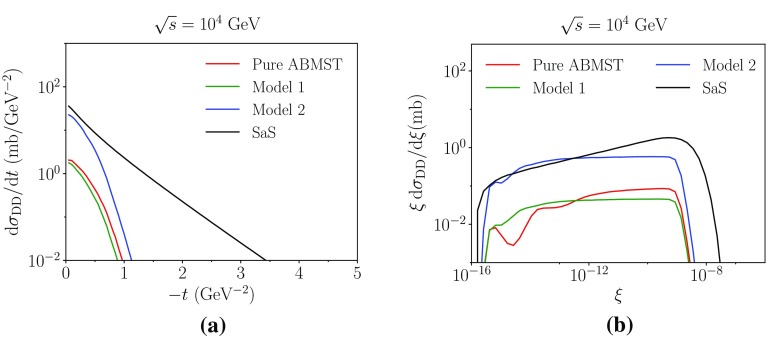
Some of the DD models available in Pythia 8. In **a**, **b** we show the differential cross section as a function of *t* and $\xi =\xi _1\xi _2$, respectively

**Fig. 10:**
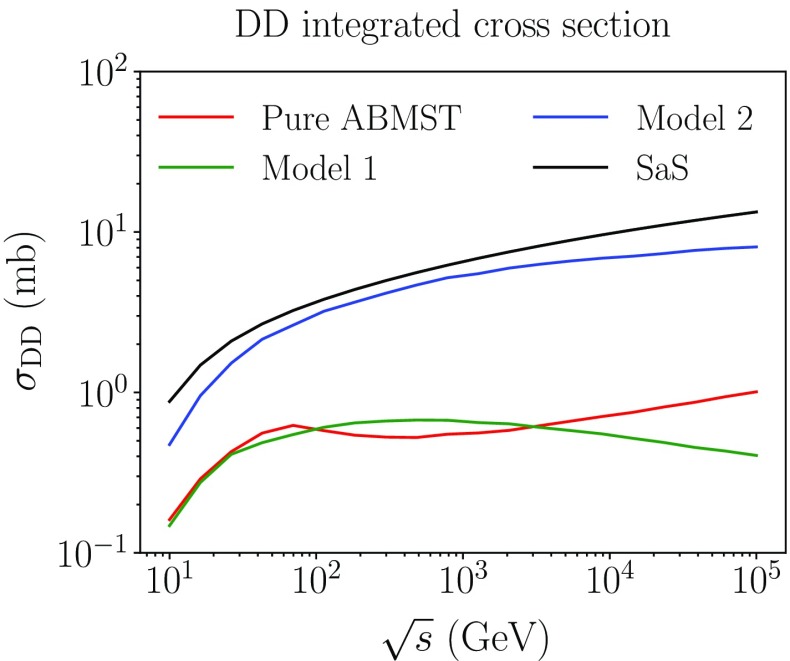
The integrated double diffractive cross section as a function of energy of some of the models available in Pythia 8

Figure 9a shows the *t* spectrum of the different models compared to the SaS model. It is evident that three models vanish faster than the SaS model. This is a result of the modest falloff of the elastic *t*-spectrum in ABMST, as this affects the double diffractive slope less than a sharply falling elastic *t*-spectrum in SaS, through the relation $B_{XY}=B_{AY} + B_{XB} - B_{\mathrm {el}}$. Figure 9b shows the differential cross section as a function of $\xi =\xi _1\xi _2$. Here, the ABMST models show an approximate $1/\xi$-behaviour, while the SaS model indicates a $1/\xi ^{(1+p)}$ behaviour with $p>0$, favouring high-mass diffractive systems. The results of these effects are visible in the integrated cross section, Fig. 10, where both “Pure ABMST” and “Model 1” are significantly suppressed compared to the SaS model. The scaled version, “Model 2”, gives more reasonable estimates of the cross sections, around 10 mb at LHC energies, but because of the choice of the power, $p=0.1$, in the scaling, it does not rise as steeply as the SaS prediction. Similarly to the single-diffractive case, the SaS model predicts slightly larger cross sections than measured, so one might expect that the scaling chosen in Model 2 could be more in agreement with measurements.

## Central diffractive cross sections

The central diffractive framework has long been neglected in general-purpose event generators. Dedicated event-generators exist for exclusive central diffractive processes, such as SuperChic [47] and ExHume [48], but these only work with a limited set of final states. Pythia 8 provides a description for inclusive high-mass central diffraction, but does not provide any such description for the exclusive processes. As stated earlier, we stress that the framework has not been tuned and thus is not to be trusted too far.

In this work we wish to extend the present description of central diffraction to include the high-mass description of the ABMST model. We have not made any attempt to include any low-mass resonances of central diffraction, as some of these are still not well established. The low-mass resonances used in ABMST are baryonic resonances, hence they cannot be extended to the central diffractive framework, as one expects scalar mesons, possibly scalar glueballs, to be produced in the collision of two Reggeons. Future work would be to extend the model to such low-mass resonances, e.g. by including a low-mass resonance description similar to what has been developed in [49]. There the central exclusive production of a pion pair is considered and data is used to fit a model of the scalar resonances using complex Breit–Wigner shapes. Lacking a model for all such exclusive states, and since some of the resonances and their decays still are not experimentally under control, we have decided not to include any of the low-mass states in this framework.

The new central diffractive cross section presented here is again mainly based on the ABMST single-diffractive model. By examining the rapidity of the different components in the central diffractive system, one obtains the following relations,

38 $$\begin{aligned} \varDelta y_{\mathrm {tot}}= & {} \ln \frac{s}{s_0},\quad \varDelta y_X = \ln \frac{\xi _1\xi _2\,s}{s_0},\nonumber \\ \varDelta y_1= & {} \ln \frac{1}{\xi _1},\quad \varDelta y_2 = \ln \frac{1}{\xi _2}, \end{aligned}$$

where $M_X^2=\xi _1\xi _2s$, $\varDelta y_X$ is the rapidity span of the diffractive system *X*, and $\varDelta y_{1,2}$ are the sizes of the two rapidity gaps. Thus, after some algebra, we obtain a central diffractive cross section of the form

39 $$\begin{aligned} \frac{{\mathrm d}^4\sigma _{\mathrm {CD}}}{{\mathrm d}\xi _1{\mathrm d}\xi _2{\mathrm d}t_1{\mathrm d}t_2}= & {} \frac{1}{256\pi ^3}\sum _{ijk} \left( \frac{s}{s_0}\right) ^{\alpha _k(0) - 1}\nonumber \\&\times g_{i{\mathrm p}}^2(t_1)g^{ii}_k(t_1) (\xi _1)^{\alpha _k(0)- 2\alpha _i(t_1)}\nonumber \\&\times g_{j{\mathrm p}}^2(t_2)g^{jj}_k(t_2) (\xi _2)^{\alpha _k(0)- 2\alpha _j(t_2)}\nonumber \\ \frac{{\mathrm d}^4\sigma _{\mathrm {CD}}}{{\mathrm d}\xi _1{\mathrm d}\xi _2{\mathrm d}t_1{\mathrm d}t_2}= & {} \frac{1}{\pi }\sum _{k}\frac{1}{g_{k{\mathrm p}}^2(0)} \left( \frac{s}{s_0}\right) ^{1 - \alpha _k(0)}\nonumber \\&\times \frac{{\mathrm d}^2\sigma _{\mathrm {HM}}}{{\mathrm d}t_1{\mathrm d}\xi _1} \frac{{\mathrm d}^2\sigma _{\mathrm {HM}}}{{\mathrm d}t_2{\mathrm d}\xi _2}\nonumber \\\rightarrow & {} \frac{1}{\pi } \frac{1}{g_{\mathbb {P}{\mathrm p}}^2(0)}\left( \frac{s}{s_0}\right) ^{1 - \alpha _{\mathbb {P}}(0)} \frac{{\mathrm d}^2\sigma _{\mathrm {HM}}}{{\mathrm d}t_1{\mathrm d}\xi _1} \frac{{\mathrm d}^2\sigma _{\mathrm {HM}}}{{\mathrm d}t_2{\mathrm d}\xi _2}\nonumber \\= & {} \frac{1}{\pi }\frac{{\mathrm d}^2\sigma _{\mathrm {HM}}}{{\mathrm d}t_1{\mathrm d}\xi _1} \frac{{\mathrm d}^2\sigma _{\mathrm {HM}}}{{\mathrm d}t_2{\mathrm d}\xi _2} \, / \, \sigma _{\mathrm {tot}}, \end{aligned}$$

where the high-energy limit is taken in the second step and recognised as the total cross section [3]. Similar arguments on the validity of Eq. (39) applies as for the validity of Eq. (36), i.e. Eq. (39) is only valid in the high-energy limit, where the Pomeron term dominates. In practise, however, the expression is used over the entire energy range, using the sum of both the soft and the hard Pomeron term from the total cross section. A scaling factor similar to the scaling for single and double diffraction can be applied, to compensate for the approximations, and the same non-vanishing gap suppression can be applied as in the single-diffractive framework. Finally, a minimal central diffractive slope can also be applied.

**Fig. 11:**
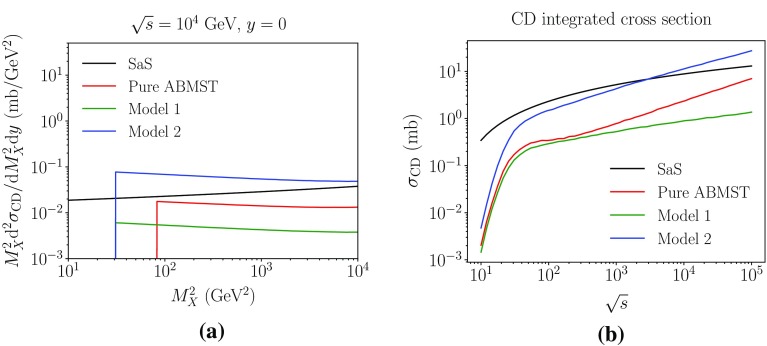
Some of the CD models available in Pythia 8. In **a** we show the mass of the diffractive system produced at central rapidity and in **b** the integrated cross section as a function of energy

Similar to the double diffractive framework, the central diffractive framework will depend on the choice of single diffractive framework, thus several options exist. Figure 11 shows three choices of models with the same name conventions as used in the double diffractive framework. Note, however, that the *t* spectrum is not shown, as this is exactly that of the single diffractive model. The mass of the diffractive system is shown in Fig. 11a, where the sharp cut at $M_X=M_{\mathrm {cut}}$ is present for all ABMST variants. The SaS model has a similar sharp cutoff, but at $M_X=1$ GeV. Lacking both model and data in the low-mass region, the cut allows for a clear distinction between what is included and not, albeit being unphysical.

Figure 11b shows the integrated cross section as a function of energy. Here all ABMST models lie below the SaS prediction, although “Model 2” exceeds it at around LHC energies. The lack of a low-mass model is evident at low energies ($\sqrt{s}<30$ GeV), where all three models decrease rapidly. In this energy-range the low-mass states make up a large part of the cross section, hence should not be neglected.

## Results

In this section the models are confronted with more recent LHC data. Several experiments have performed measurements on integrated cross sections and diffractive fractions, but not many provide results on differential distributions. We focus on the analyses available in Rivet [50], where only two analyses provide differential results. First we provide a discussion of the available data and the tuning prospects, and end with results obtained with the SaS model, the CSCR model and the ABMST models.

### The 7 TeV LHC data and tuning prospects

In 2012 and 2015 ATLAS [51] and CMS [52] presented results on 7 TeV events with rapidity gaps. Both experiments measure all particles with transverse momenta larger than 200 MeV in pseudo-rapidity ranges of $|\eta | < 4.9 \, (4.7)$ for ATLAS (CMS), and define the measured gap $\varDelta \eta _F$ as the largest distance between either detector edge and the particle nearest to it. The two experiments, however, obtain different results for the shape of the distribution.

**Fig. 12:**
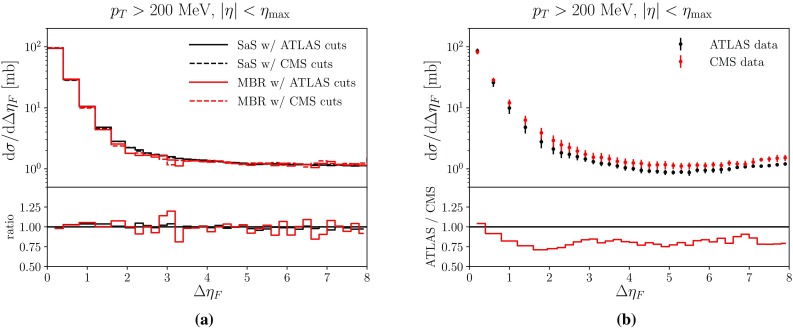
**a** The SaS and MBR models using either ATLAS or CMS cuts along with the ratio of ATLAS to CMS cuts for both models. **b** The ATLAS [51] and CMS [52] data along with the ratio of ATLAS to CMS data, showing significant differences in the entire range

In Fig. 12a, we show the results obtained with default Pythia 8 using the SaS model and the MBR model when comparing to either the ATLAS or CMS Rivet analyses. Both models are shown, since ATLAS uses the SaS model for unfolding, while CMS uses the MBR one, but model agreement is sufficiently close that unfolding differences should not be an issue. Further, from Fig. 12a it is evident that the different experimental $\eta$ cuts gives at most a 5% effect on either model. This does not account for the approximately 25% difference seen in data, see Fig. 12b. A tune to both datasets will not be able to describe either perfectly, as they so clearly disagree. Experiment-specific tunes would likely improve the description of that particular dataset, hence worsening the description of the other. As we cannot decide which of the two is the preferred one, we instead aim for the middle ground.

Besides the above mentioned datasets measurements of the inelastic and diffractive cross sections have been performed by both ATLAS, CMS and ALICE. We include the following measurements: the inelastic cross section from ATLAS 2011 [53], the inelastic cross section from CMS 2012 [54] and the inelastic and diffractive cross sections from ALICE 2012 [55].

**Fig. 13:**
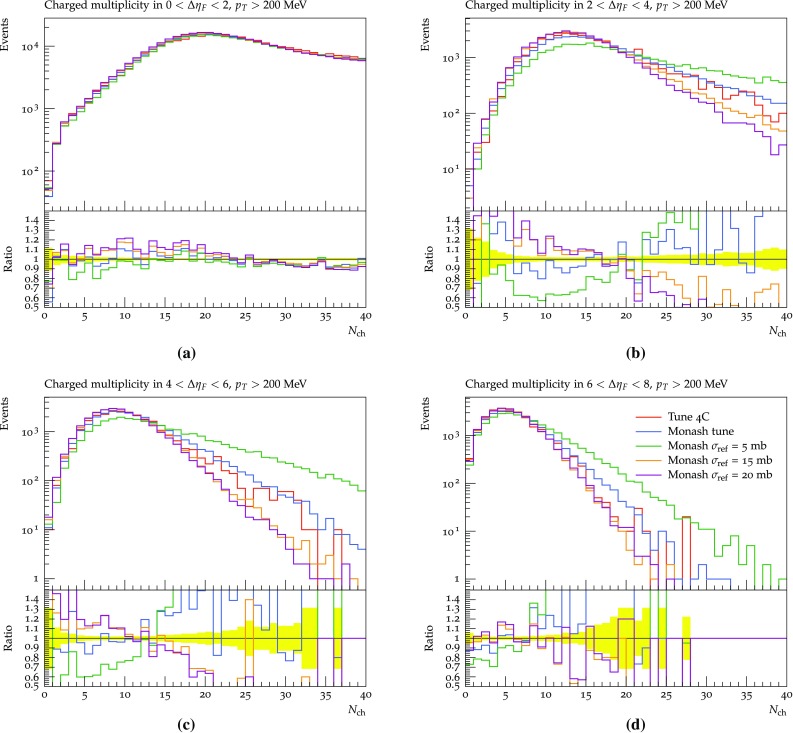
The effects of changing the reference $\mathbb {P}{\mathrm p}$ cross section on the charged multiplicity distribution using the SaS model at 7 TeV in the four gap ranges: 0–2 (**a**), 2–4 (**b**), 4–6 (**c**) and 6–8 (**d**)

**Fig. 14:**
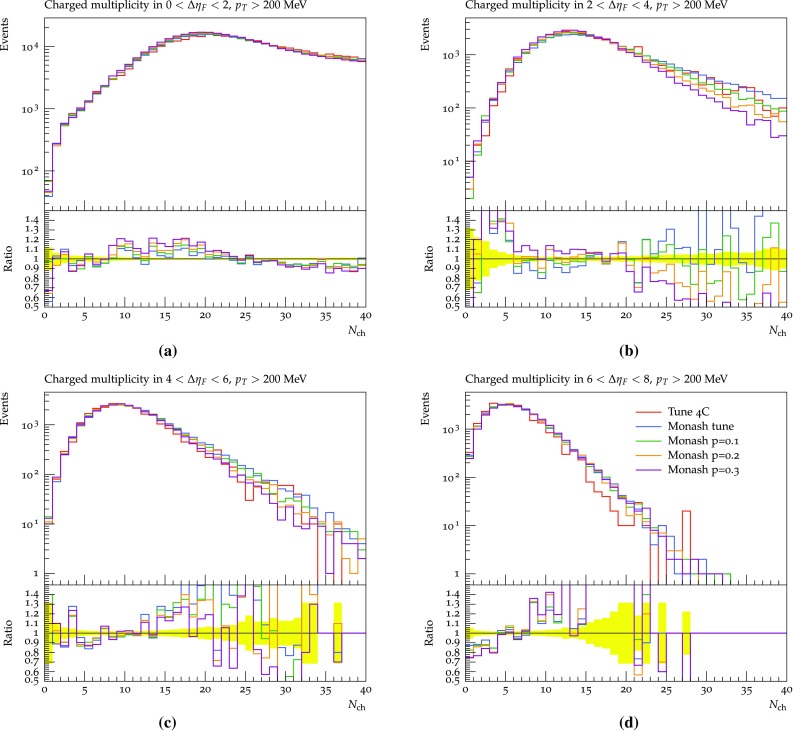
The effects of changing the power of the mass dependence in the $\mathbb {P}{\mathrm p}$ cross section on the charged multiplicity distribution using the SaS model at 7 TeV in the four gap ranges: 0–2 (**a**), 2–4 (**b**), 4–6 (**c**) and 6–8 (**d**)

None of the datasets available in the Rivet framework are able to constrain the parameters related to the hadronic event properties. This includes both the low-to-high-mass transition probability parameters as well as the parameters of the non-perturbative and perturbative description of the evolution of the diffractive system. In particular, the non-perturbative description is left as is in this study, while the effects of changing the $\mathbb {P}{\mathrm p}$ cross section is shown in Figs. 13 and 14. This cross section determines the amount of multiparton interactions activity in a high-mass diffractive event, and thereby e.g. the charged multiplicity distribution. It is interesting because of discrepancies between uncorrected ATLAS data and the Pythia 4C tune (Fig. 3a–d in [51]). A direct comparison cannot be made, since the ATLAS distributions show the number of electromagnetic clusters rather than that of charged particles, but the two clearly are related.

Figures 13 and 14 show the effects of changing the $\mathbb {P}{\mathrm p}$ cross section on the charged particle distributions in the different $\varDelta \eta _F$ bins compared to the 4C tune. In [51] the 4C tune generally was seen to undershoot the low cluster-multiplicities, while overshooting the mid to high cluster multiplicities. In the highest $\varDelta \eta _F$ bin, dominated by the diffractive events, Tune 4C undershoots both the low- and high-multiplicity activity. Reducing the $\mathbb {P}{\mathrm p}$ cross increases the multiplicity, and vice versa. Thus, to describe the high-multiplicity events, a smaller $\mathbb {P}{\mathrm p}$ cross section would be preferred. This could be compensated by allowing the perturbative description to go below $M_X=m_{\mathrm {min}}=10$ GeV, thus allowing slightly more activity in low-mass systems, possibly increasing the number of low-multiplicity events. The effects of including a mass dependence in the $\mathbb {P}{\mathrm p}$ cross section is seen in Fig. 14. A parametrization has been chosen as $\sigma _{\mathbb {P}{\mathrm p}}^{\mathrm {eff}}(M_X) = \sigma _{\mathbb {P}{\mathrm p}}^{\mathrm {ref}} \, (M_X / M_{\mathrm {ref}})^p$, with $M_{\mathrm {ref}} = 100$ GeV. Here, an increase of *p* slightly decreases the high-multiplicity region, albeit more subtly than with an increase of the $\mathbb {P}{\mathrm p}$ cross section. Recall that the mass of the diffractive system is related to the collision energy, such that a value of $p\sim 0.2-0.3$ is not unreasonable, corresponding to a rise of the cross section with energy of $s^{0.1}-s^{0.15}$.

**Fig. 15:**
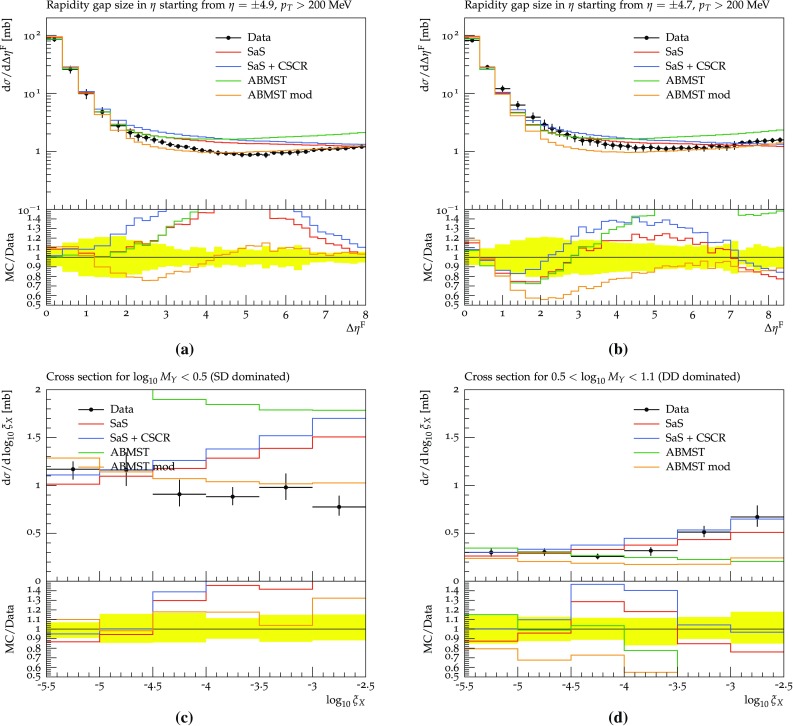
The cross section as a function of gap size for the default SaS model, the SaS+CSCR model and the untuned ABMST models compared to ATLAS [51] (**a**) and CMS [52] (**b**) data. The cross section as a function of $\mathrm {log}_{10}\xi _X$ in a single-diffraction dominated region (**c**) and double diffraction dominated region (**d**) compared to CMS [52] data

A full study of particle production in diffractive events with Pythia 8, Herwig 7 [56, 57], Sherpa [58, 59] and Phojet [60] could provide further valuable information on the hadronic event properties of diffractive systems, as the generators differ in how they describe such production. The effects of colour reconnection in a diffractive system is also of interest, as the amount of “accidental” gaps could be constrained in these systems, if one assumes that the CR scheme is the same in both diffractive and non-diffractive systems. At present we leave the $\mathbb {P}{\mathrm p}$ cross section as is at 10 mb, and show the results with the models presented so far in Fig. 15.

The bulk of the cross section arises from nondiffractive events. These tend to only give rise to small rapidity gaps, as the phase space is more or less evenly filled by multiparton interactions. Gaps of intermediate or large size can occur, however, e.g. by colour reconnection between the partons [9, 61]. The default Pythia CR framework has been designed to avoid accidental gaps, so as to keep a clean separation between diffractive and nondiffractive topologies. In other models, e.g. the CSCR one [18], the colour reshuffling tends to give somewhat larger probability for intermediate gaps. A combination of the CSCR model and the default SaS diffractive setup then results in too large a cross section in the intermediate-gap range, cf. Fig. 15a, b.

Diffractive events are more likely to give rise to intermediate to large gaps. Hence, depending on colour-reconnection model used, they will dominate from gap sizes of approximately two and larger. The size of the gap is closely connected to the mass of the diffractive system. Thus a model with a ${\mathrm d}M_X^2/M_X^2$ ansatz, like the SaS one (modulo some corrections), will give an approximately flat distribution of measured gap sizes. This can be modified by the recent inclusion of the mass-correction factor $\epsilon _{\mathrm {SaS}}$, which introduces an additional $1/M_X^{2\epsilon _{\mathrm {SaS}}}$ factor to the differential model. Depending on the sign of $\epsilon _{\mathrm {SaS}}$, it will either increase or decrease the high-mass cross section. In both the ATLAS and CMS datasets an increase of the large-gap cross section is seen. Thus we expect a positive sign for $\epsilon _{\mathrm {SaS}}$, as this will enhance the activity at low masses. For simplicity, adding the mass correction will not affect the integrated diffractive cross section.

The ABMST models show slightly better agreement with the shape of the rapidity gap distributions, although the original ABMST model overshoots both datasets. This was to be expected, as the model had trouble with the increase of the single diffractive cross section at LHC energies. The modified version of the ABMST model shows very nice agreement with both datasets, except for an undershoot of the high-mass region of the double-diffractive-dominated region in Fig. 15d. This behaviour closely correlates with the flatness of the $\xi {\mathrm d}\sigma /{\mathrm d}\xi$-spectrum, Fig. 9b. Both the ABMST models have a mass spectrum shape comparable to data in the single-diffraction-dominated region, unlike the SaS model, which overshoots the high-mass systems.

### The tuned models

The tunes provided here are performed with the Professor framework [62], varying the high-mass diffractive parameters given in Table 1. All non-diffractive parameters are left at their default values, as given by the Monash tune [63], except for the CSCR-specific changes in that setup. The H1 leading order Pomeron PDF [27] is used in all the tunes.

**Table 1:** The parameters used in the tunes for the different models

	$\epsilon$	$\alpha '$	$\sigma _{\mathrm {SD}}^{\mathrm {max}}$	$\sigma _{\mathrm {DD}}^{\mathrm {max}}$	$\sigma _{\mathrm {CD}}^{\mathrm {max}}$	$\epsilon _{\mathrm {SaS}}$
SaS	0.06	0.4	22.31	39.83	0	0
SaS+CSCR	0.15	0.26	20.81	13.13	0	0
SaS+$\epsilon _{\mathrm {SaS}}$	0.04	0.30	24.78	52.49	0	0.08

	$k_{\mathrm {SD}}$	$k_{\mathrm {DD}}$	$k_{\mathrm {CD}}$	$p_{\mathrm {SD}}$	$p_{\mathrm {DD}}$	$p_{\mathrm {CD}}$
ABMST	0.58	2.45	1.0	0	0.05	0.03
ABMST modified	0.92	1.72	1.38	0	0.1	0.04

**Fig. 16:**
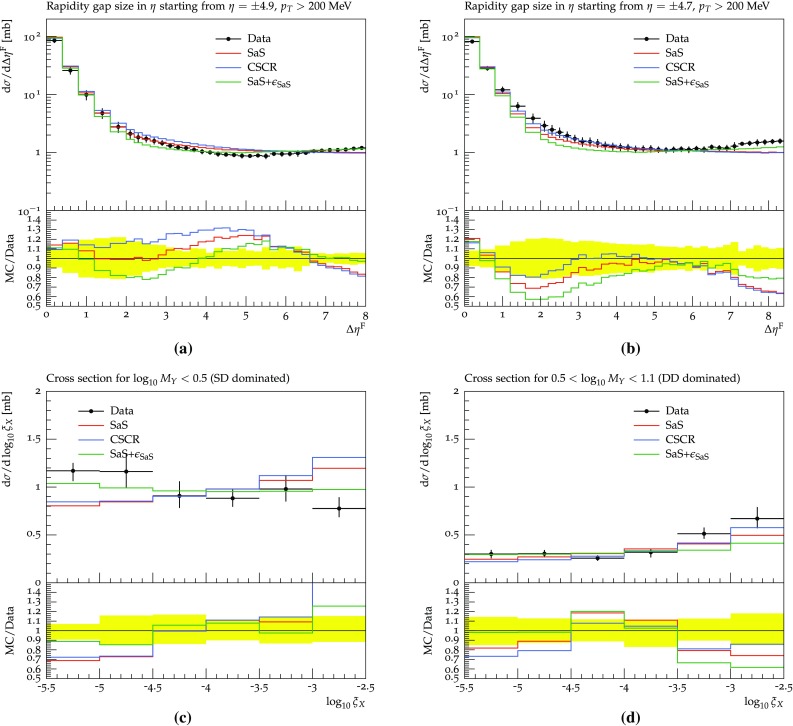
The cross section as a function of gap size for the three SaS-based models compared to ATLAS [51] (**a**) and CMS [52] (**b**) data. The cross section as a function of $\mathrm {log}_{10}\xi _X$ in a single-diffraction dominated region (**c**) and double diffractive dominated region (**d**) compared to CMS [52] data

Figure 16 shows the three SaS-based models tuned to the above-mentioned data. Neither of the three models are able to describe the shape of the gap data perfectly, Fig. 16a, b. The tune has decreased the amount of activity in the mid- to large-gap region by a decrease of the $\sigma _i^{\mathrm {max}}$ values used in Eq. (9). The inclusion of $\epsilon _{\mathrm {SaS}}$ has shifted some of the activity from intermediate-gaps to larger ones, while keeping the integrated cross section fixed. Unfortunately this is at the expense of an undershoot in the transition region $\varDelta \eta ^{\mathrm {F}} \sim 2$ between diffractive and nondiffractive topologies. This is the region where CSCR does better, so a combination of CSCR with an $\epsilon _{\mathrm {SaS}} > 0$ could provide a flatter MC/data distribution in Fig. 16a, b.

For the mass spectra measured by CMS, Fig. 16c, d, evidently only the SaS+$\epsilon _{\mathrm {SaS}}$ model is able to describe the single-diffraction-dominated mass spectrum, whereas it undershoots the high-mass double diffraction region since, relative to the original SaS model, it has shifted some of the high-mass activity to lower masses.

**Fig. 17:**
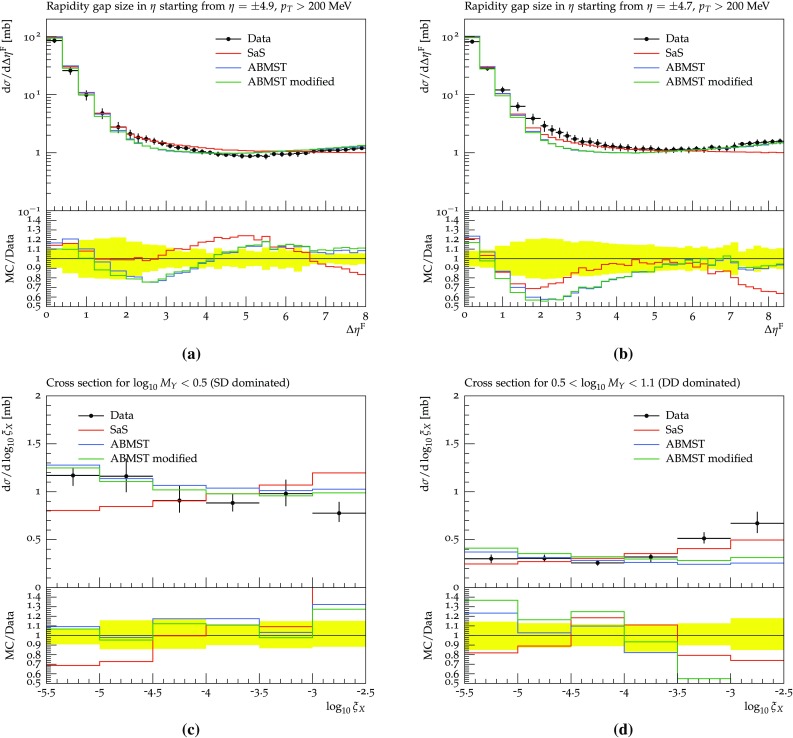
The cross section as a function of gap size for the two ABMST-based models compared to ATLAS [51] (**a**) and CMS [52] (**b**) data. The cross section as a function of $\mathrm {log}_{10}\xi _X$ in a single-diffraction dominated region (**c**) and double diffractive dominated region (**d**) compared to CMS [52] data. For reference the tuned SaS model is also shown

Figure 17 shows the tuned ABMST models. The tune has a hard time improving the modified ABMST model, as this gave a good agreement with data already to begin with. The original ABMST model, however, is significantly improved by rescaling, and is now very similar to the modified ABMST model developed in this paper. Note that the mass spectrum of the single-diffraction-dominated region, Fig. 17c, shows the proper shape, while the double-diffraction-dominated one, Fig. 17d, seems to overestimate the low-mass region and underestimate the high-mass one. This is a result of the reduction of the vanishing-gap topologies of double-diffractive systems, that has been kept unchanged in this tune. Combining the ABMST models with the CSCR model has potential also here, as the ABMST models underestimate data in the intermediate gap range, cf. Fig. 17a, b.

**Fig. 18:**
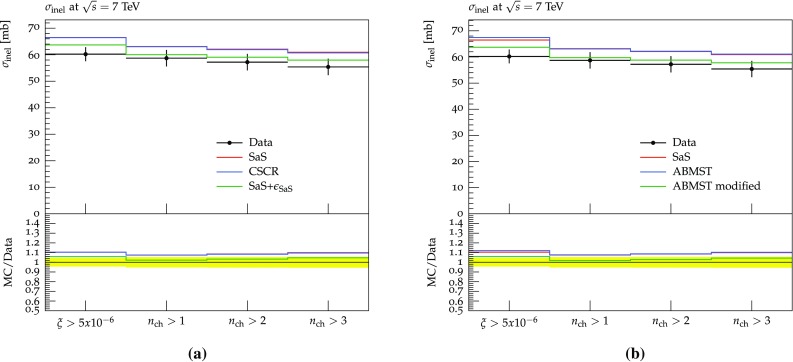
The inelastic cross section as a function of method described in the text compared to CMS [54] data

Figure 18 shows the CMS inelastic cross section obtained with two different approaches. One uses forward calorimetry ($3< |\eta | < 5$), to measure protons with fractional momentum loss greater than $\xi > 5\cdot 10^{-6}$, corresponding to everything but low-mass diffractive systems ($M_X>16$ GeV). The other uses the central tracker, requiring either one, two or three tracks. The SaS+$\epsilon _{\mathrm {SaS}}$ and the modified ABMST models perform better than the others, with a maximum 5% deviation from CMS data. The SaS and the CSCR models has the same model for diffractive systems, and hence it is not expected that these differ in the measured inelastic cross section. With the SaS+$\epsilon _{\mathrm {SaS}}$ model, however, some of the activity has been shifted to lower diffractive masses, resulting in a lower inelastic cross section. For ABMST, the reduction of the high-mass systems in the modified model results in a reduction of the inelastic cross section relative to the original one.

Table 2 shows the integrated cross sections obtained with the ALICE and ATLAS 2011 analyses mentioned above. The ALICE results have been obtained for $M_X<200$ GeV ($\xi < 0.0008$) for single diffraction, for gap sizes larger than $\varDelta \eta >3$ for double diffraction, and with a van der Meer scan using diffractive events adjusted to data for the inelastic cross section. In the Rivet analysis, this corresponds to at least two tracks in the final state, i.e. effectively without any experimental cuts and hence returning the generator-level cross section. The SaS+$\epsilon _{\mathrm {SaS}}$ model gives a better prediction for the single diffractive data, because of the increased low-mass cross section. The CSCR model predicts a larger double diffractive cross section, because of the larger probability for “accidental” gaps. The inelastic cross section, however, is the same for all three SaS-based models when compared with the ALICE data, as all have the same generator-level integrated cross section. In the ATLAS measurement of the inelastic cross section (for $\xi >5\cdot 10^{-6}$) the SaS+$\epsilon _{\mathrm {SaS}}$ model predicts a lower inelastic cross section, again because of the larger low-mass cross section.

Both the ABMST models give larger single-diffractive cross sections than SaS, having improved in the low-mass region. But both underestimate the double-diffractive cross section as they both underestimate the medium-sized gaps, compared with SaS and data. An addition of the CSCR model would be likely to improve this prediction. The ABMST models predict the same inelastic cross section for ALICE, since the generator-level inelastic cross section is the same for the two models. They differ for the ATLAS analysis, again because of the reduced high-mass systems of the modified ABMST model.

**Table 2:** The integrated cross section obtained with the three aforementioned Rivet analyses for the tuned models. For ALICE [55], the SD cross section is for $M_X < 200$ GeV, the double diffractive for gaps larger than 3, the inelastic using a van der Meer scan using diffractive events adjusted to data. The ATLAS [53] inelastic cross section is for $\xi > 5\cdot 10^{-6}$

	$\sigma _{\mathrm {SD}}$ (mb)	$\sigma _{\mathrm {DD}}$ (mb)	$\sigma _{\mathrm {inel}}$ (mb)	$\sigma _{\mathrm {inel}}$ (mb)
	(ALICE)	(ALICE)	(ALICE)	(ATLAS)
Data	14.9 ± 5.90	9.00 ± 2.60	73.20 ± 5.28	60.33 ± 2.10
SaS	6.13 ± 0.01	5.72 ± 0.01	71.06 ± 0.02	66.48 ± 0.02
SaS + CSCR	6.15 ± 0.01	6.19 ± 0.01	71.06 ± 0.02	66.43 ± 0.02
SaS + $\epsilon _{\mathrm {SaS}}$	7.98 ± 0.01	5.62 ± 0.01	71.06 ± 0.02	63.69 ± 0.02
ABMST	7.24 ± 0.01	4.69 ± 0.01	71.62 ± 0.02	67.44 ± 0.02
ABMST mod	9.41 ± 0.01	5.09 ± 0.01	71.62 ± 0.02	63.72 ± 0.02

In general, however, all models fail to describe the measured integrated cross sections, although some of the more sophisticated models do improve in some respects. Similarly, it seems that neither of the models describe well the transition from a non-diffractive-dominated region to a diffraction-dominated one. Including a colour-reconnection model that allows for larger gaps in the non-diffractive events, like CSCR, is likely to improve the description in the mid-sized-gap range, if combined with a model that predicts a lower diffractive cross section there, like the ABMST models and SaS+$\epsilon _{\mathrm {SaS}}$. The overall question of how to combine the descriptions of non-diffractive and diffractive topologies, however, will still exist even if the CR model “accidentally” (i.e. by “accidental” gaps) improves the description of data. All this highlights our still limited understanding of nonperturbative QCD, which forces us to work with models e.g. rooted in Regge theory. This may be good enough for an overall understanding, but still not for a precise reproduction of all relevant data.

## Conclusions

In this paper we provide an updated description of the cross sections and hadronic event shapes in the event generator Pythia 8. The update has been required since the first results appeared from the LHC experiments, showing significant discrepancies between the models provided by Donnachie and Landshoff for the total cross section, as well as the elastic and diffractive cross sections by Schuler and Sjöstrand. By chance the DL undershooting of the total cross section and the SaS undershooting of the elastic cross section partly cancel in the inelastic cross section. Further to that, the SaS overshooting of the diffractive cross sections gave rise to a reasonable agreement between Pythia 8 and LHC measurements on the observable non-diffractive cross section, which is the relevant one for many of the measurements performed at the LHC. Thus, in spite of these shortfalls, the default Pythia 8 cross sections usually were good enough, notably when diffractive cross sections had been reduced somewhat (Eq. 9).

The discrepancies became largely evident with the precision measurements of the elastic and total cross sections performed by both TOTEM and ATLAS+ALFA. Here the exponential shape of the *t* spectrum in Pythia 8 is too simplistic, and other models have to be used for comparisons. Some of these models have now been implemented into Pythia 8, thereby providing a more sophisticated framework for elastic scattering and total cross sections.

For diffractive topologies the precision is less. The studies are marred by non-diffractive events mimicking diffractive ones, and vice versa, making the explicit distinction between the various diffractive and non-diffractive event topologies hard. The possibility of tagging the elastically scattered protons would greatly improve the separation of the samples, but so far no analyses on diffraction with tagged protons have appeared from CMS + TOTEM or ATLAS + ALFA. Thus we are left with measurements only using the central general-purpose detectors. Unfortunately these do not give fully consistent answers. Notably the CMS and ATLAS rapidity-gap measurements disagree in the diffraction-dominated region, making it hard to compare models with data. Lacking any further guidance, we have here aimed for a middle ground between the two data sets.

The situation is even worse for of hadronic event shapes. Single diffractive data is available for very low energies, most of which goes into the ABMST model, but rather little for higher energies. This means that, even if integrated cross sections were provided for diffractive topologies from the LHC experiments, no constraints are put on the internal structure of diffractive systems. The ansatz of Pythia 8, that the diffractive system properties are similar to those of non-diffractive events, could be wrong. A future study of these event shapes, and of the different strategies underlying commonly used event generators, would help provide a guideline what would be interesting distributions to see measured at the LHC.

In conclusion, we provide an updated and extended framework for elastic and diffractive topologies, as well as an update for all parts of the total cross section. We rely on previous work provided by several other authors, but have corrected and extended the models where need be. Each of the models have been tuned to available data, thus providing an upgrade of the already present models in Pythia 8. We have discussed some of the consequences of different approaches for creating rapidity gaps, such as the CSCR model, and how this affects the predictions for LHC. Still, the lack of data or the discrepancies of present data, leaves us with imperfect descriptions and predictions, in particular for diffraction. The situation may be “good enough” for current needs, but will hopefully improve with new data in the future. At present we are not able to decide which model is “the better one” for diffraction, but in the case of total and elastic cross section the new models, COMPAS and ABMST, offer an improved description as compared to the SaS model. As the COMPAS model offers no description of diffraction, we propose to use the ABMST model for total and elastic cross section and the modified ABMST model for diffraction, with the tuned parameters as provided in this paper. We expect to change the default behaviour in the next Pythia release.

Foreseeable further work could include a low-mass description for central diffractive topologies, possibly modelling the resonances present there. Other work would be an extensive study of the diffractive event shapes as discussed above. A study on eikonalisation aspects, e.g. of events with both diffractive and nondiffractive Pomeron exchanges, could also provide more insight on both cross sections and event topologies. Finally, the diffractive framework could be extended also to other processes, such as $\gamma {\mathrm p}$ and $\gamma \gamma$ collisions.
